# Impact of mHealth Chronic Disease Management on Treatment Adherence and Patient Outcomes: A Systematic Review

**DOI:** 10.2196/jmir.3951

**Published:** 2015-02-24

**Authors:** Saee Hamine, Emily Gerth-Guyette, Dunia Faulx, Beverly B Green, Amy Sarah Ginsburg

**Affiliations:** ^1^University of WashingtonSeattle, WAUnited States; ^2^PATHSeattle, WAUnited States; ^3^Group Health CooperativeGroup Health Research InstituteSeattle, WAUnited States

**Keywords:** telemedicine, mHealth, mobile health, patient compliance, patient adherence, chronic disease, diabetes mellitus, cardiovascular diseases, lung diseases

## Abstract

**Background:**

Adherence to chronic disease management is critical to achieving improved health outcomes, quality of life, and cost-effective health care. As the burden of chronic diseases continues to grow globally, so does the impact of non-adherence. Mobile technologies are increasingly being used in health care and public health practice (mHealth) for patient communication, monitoring, and education, and to facilitate adherence to chronic diseases management.

**Objective:**

We conducted a systematic review of the literature to evaluate the effectiveness of mHealth in supporting the adherence of patients to chronic diseases management (“mAdherence”), and the usability, feasibility, and acceptability of mAdherence tools and platforms in chronic disease management among patients and health care providers.

**Methods:**

We searched PubMed, Embase, and EBSCO databases for studies that assessed the role of mAdherence in chronic disease management of diabetes mellitus, cardiovascular disease, and chronic lung diseases from 1980 through May 2014. Outcomes of interest included effect of mHealth on patient adherence to chronic diseases management, disease-specific clinical outcomes after intervention, and the usability, feasibility, and acceptability of mAdherence tools and platforms in chronic disease management among target end-users.

**Results:**

In all, 107 articles met all inclusion criteria. Short message service was the most commonly used mAdherence tool in 40.2% (43/107) of studies. Usability, feasibility, and acceptability or patient preferences for mAdherence interventions were assessed in 57.9% (62/107) of studies and found to be generally high. A total of 27 studies employed randomized controlled trial (RCT) methods to assess impact on adherence behaviors, and significant improvements were observed in 15 of those studies (56%). Of the 41 RCTs that measured effects on disease-specific clinical outcomes, significant improvements between groups were reported in 16 studies (39%).

**Conclusions:**

There is potential for mHealth tools to better facilitate adherence to chronic disease management, but the evidence supporting its current effectiveness is mixed. Further research should focus on understanding and improving how mHealth tools can overcome specific barriers to adherence.

## Introduction

Chronic diseases are the most common causes of death and disability worldwide [1]. Chronic disease management often requires a long-term care plan. Adherence to chronic disease management is critical to achieving improved health outcomes, quality of life, and cost-effective health care [1]. A World Health Organization review of adherence behaviors noted that, “increasing adherence may have a greater effect on health than improvements in specific medical therapy” [2]. With an average adherence rate of only 50% among patients with chronic diseases, non-adherence is a serious challenge to chronic disease management [3]. The extent of non-adherence is even higher in developing countries [3-5]. The long-term nature and frequent need for continuous monitoring in chronic disease management gave rise to early developments in telehealth and telemonitoring. These innovations, which seek to improve chronic disease management and prevent death and disability, are improved by ongoing technological advancements.

One such advancement is mHealth—health care and public health practice supported by mobile devices [6]. Close to three-quarters of the world’s population has access to a mobile phone with increasingly powerful technical capacities [7]. More than 6.9 billion mobile subscriptions were in use as of May 2014, 5.4 billion of which were in developing countries [8]. Based on their popularity, availability, portability, and technological capacity, mobile phones and mHealth have enormous potential to impact chronic disease management around the globe. A World Health Organization survey of global mHealth initiatives published in 2011 reported a “groundswell” of activity in both developed and developing countries [6]. Mobile technologies such as phones and wireless monitoring devices are increasingly being used in health care and public health practice for communication, data collection, patient monitoring, and education, and to facilitate adherence to chronic disease management [6]. mHealth devices can improve service delivery and impact patient outcomes [6]. Sensors and context-awareness features allow for individualization and real-time information submission delivery. Moreover, the strong attachment people have to mobile phones and the tendency to carry them everywhere, opens up opportunities for continuous symptom monitoring and connecting patients with providers outside of health care facilities.

While the growing popularity of mHealth is evident, its impact is not. The reported impact of mHealth interventions is mixed, with studies showing modest benefits for some clinical diagnosis and management support outcomes [9,10]. Studies have shown the positive impact of mHealth on adherence-related behavior among patients with human immunodeficiency virus (HIV) and tuberculosis. For example, short message service (SMS) appointment reminders have led to an increase in attendance among children exposed to or infected with HIV in Cameroon [11]. However, criticism of mHealth includes its implementation through small pilot initiatives that address a single disease or issue in service delivery and lack of globally accepted ways to evaluate effectiveness [7]. Systematic reviews to date are indicative of mHealth’s segmented nature, which may include a single technology or a single chronic disease or a specific mHealth application [12-14]. Another review, focused on the impact of SMS interventions, found that text messaging increased adherence to antiretroviral treatment with reductions in viral load and biochemically verified smoking cessation, yet these effects were “small and of borderline clinical importance” [9]. A more comprehensive Cochrane review assessed the health impact of SMS on any type of long-term illness, but found only four comparative effectiveness trials able to address the impact of mobile services on self-management [14]. Moreover, the literature search did not go past 2009, and we are unaware of any updates.

The impact of these mHealth tools on adherence to treatment regimens may be overlooked, as mHealth promoters are eager to demonstrate their effect on clinical outcomes (eg, morbidity, mortality, and biometric markers of clinical disease). Adherence to treatment, and specifically adherence to treatment of chronic diseases, is a critical link that connects the promise of mHealth to the ultimate goal of improved clinical outcomes. This review builds the evidence base of mHealth by updating previous reviews and assessing a broad range of outcomes from usability to impact on health outcomes. This enables us to consider mHealth tools at all stages of development and gauge the effectiveness of mHealth interventions across a range of technologies and chronic diseases, many of which have overlapping treatment regimens and require similar adherence behaviors. This review aims to evaluate the effectiveness of mHealth in supporting adherence of patients to chronic disease management—which we call “mAdherence”—and the usability, feasibility, and acceptability of mAdherence tools and platforms for chronic disease management.

## Methods

### Overview

We undertook a systematic review of mHealth interventions used to facilitate adherence to chronic disease management. The chronic diseases included are diabetes mellitus (DM), cardiovascular diseases (CVDs), and chronic lung diseases (CLDs). CVDs include hypertension (HTN), coronary artery disease, and congestive heart failure. CLDs include asthma and chronic obstructive pulmonary disease (COPD). These chronic diseases were chosen based on their high global burden [15]. Our definition of mHealth was adopted from the Global Observatory for eHealth definition: “medical and public health practice supported by mobile devices” [6]. We use the term “mAdherence” to refer to any use of mHealth tools by patients and health care providers to improve adherence to chronic disease management. Given the comprehensive nature of chronic disease management, this review goes beyond defining adherence as compliance with a treatment regimen and includes a wide range of interventions, such as medication reminders, symptom monitoring, educational tools, and facilitated patient-provider communication [16].

Employing Boolean phrases, we searched PubMed, Embase, and EBSCO databases for studies that assessed the role of mAdherence in chronic disease management of DM, CVD, and CLD. MeSH terms (Medical Subject Headings) and advanced search-builder features were used for the PubMed searches. Emtree terms using the explosion function to extend the search were employed to build a multi-term query along with advanced searches in Embase. Finally, CINAHL, PsychInfo, and PsychArticles were included for searches in the EBSCO database. EndnoteWeb was used for sorting and removal of duplicates. We searched databases for articles published from 1980 through May 2014.

### Inclusion and Exclusion Criteria

We included original research published in peer-reviewed journals that evaluated mHealth tools for effect on patient adherence to chronic disease management, disease-specific clinical outcomes, and usability, feasibility, and acceptability features. mHealth interventions aimed at improving chronic disease management were included even if the research did not address adherence specifically. Usability, feasibility, and acceptability studies that focused on the design and development stages of mAdherence interventions were included as a necessary precursor to future evaluation. Studies that measured adherence included outcomes such as use of the mHealth tool for monitoring and reporting symptoms, compliance with medication regimens, and engagement in healthy behaviors. Studies that focused on clinical measures, such as hemoglobin A1c (HbA1c) or blood pressure (BP), were included, as improved clinical outcomes are the eventual goal of improving adherence and often indicate adherence to chronic disease management indirectly. Allowing for flexibility in the outcomes measured was necessary for an inclusive view of mAdherence technologies in all stages of design, development, and evaluation.

mHealth included any mobile device or service, such as mobile phones, SMS, smartphones, personal digital assistants, and devices that work on wireless technology or Bluetooth-compatible devices. These devices and services allowed patients to monitor their health, access health information, and communicate with their health care provider without requiring a wired connection to the Internet. We included interventions delivered using a Web-based platform only if it was specified that the patient accessed the service via a mobile phone or other mobile device. It was required that patients be the primary users of the mAdherence tools.

Only articles reporting that the mAdherence intervention was designed for secondary prevention targeting chronic disease patients were included. We excluded reports of studies on primary prevention among healthy or at-risk groups. We also excluded articles regarding interventions that were not tested in a sample population with clearly described methods and results. In addition, review articles, editorials, commentaries, dissertations, poster presentations, abstracts only, proposals for future studies, study protocols, and descriptive articles describing new tools but not testing them in a sample population were excluded. Publication language was restricted to English only.

### Data Extraction and Analysis

Publications were initially screened for potential inclusion based on simultaneous review of title and abstract by two reviewers. Any discrepancies were resolved by consensus among the researchers. Information including objectives, types of mobile technology used, role of mAdherence tools in chronic diseases management, setting, study sample characteristics, outcomes measured, and results reported were extracted using Microsoft Excel. Studies were organized for analysis based on the primary objective of the study and the key outcomes measured. Outcomes were organized into qualitative usability, feasibility, and acceptability of the mAdherence tool or platform among target end-users, the effect of mAdherence on patient adherence to chronic diseases management, and disease-specific clinical outcomes of the mAdherence intervention. We performed descriptive analyses of the data and summarized the findings from these studies, with emphasis on statistical results reported in randomized controlled trials (RCTs). We highlighted differences between groups when these results were available.

## Results

### Summary

**Figure 1:**
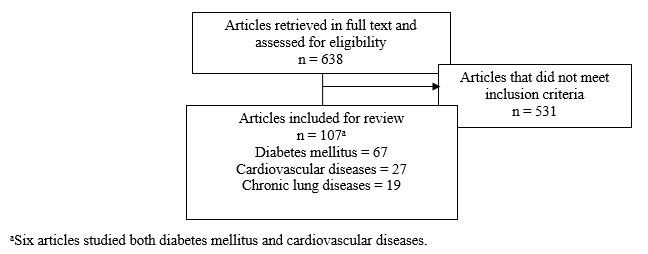
Study selection process.

In all, 638 articles were retrieved in full text and assessed for eligibility. Based on the search criteria, 531 articles were excluded. Of the excluded articles, many did not meet the study design criteria (n=225) or did not align with the definition of mHealth used here (n=199). A total of 60 articles were beyond the scope of the chronic diseases considered in this review, and 116 articles did not include any adherence component. An additional 20 articles were excluded because they were not available in English or a full text version was not obtainable despite all reasonable attempts. A total of 107 articles met all inclusion criteria. Figure 1 illustrates the selection process.

### Study Characteristics

**Figure 2:**
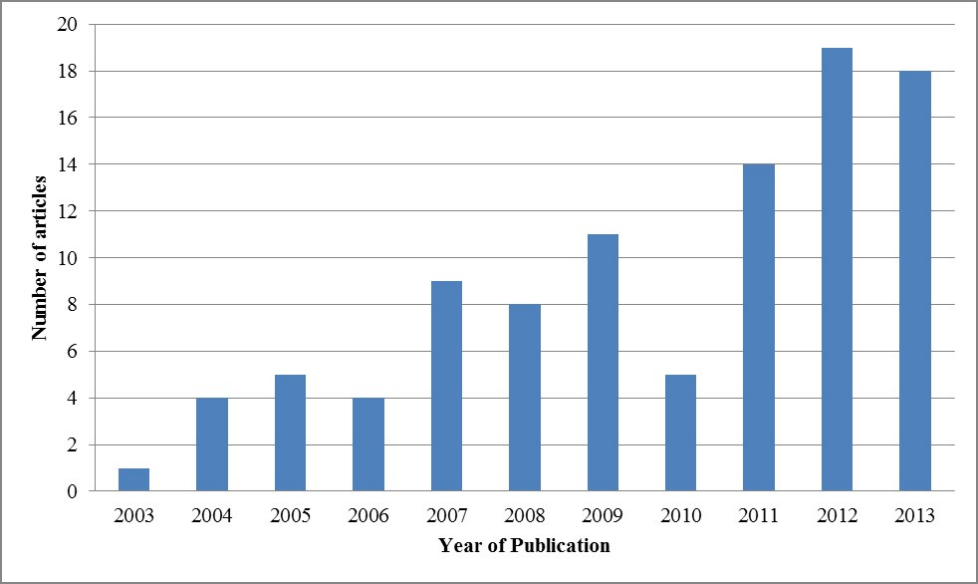
mAdherence studies published over time.

**Figure 3:**
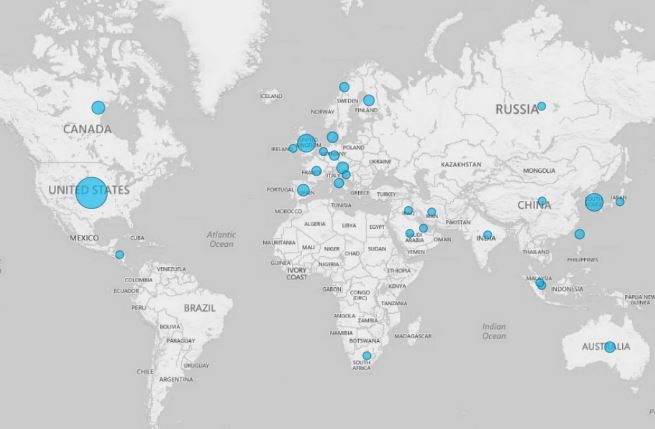
mAdherence studies published by country.

Publication years ranged from 2003 to 2014, with an overall increase in articles published more recently (Figure 2). None of the studies published before 2003 met our inclusion criteria. A total of 34.6% (37/107) of studies were conducted in the United States, followed by 10.3% (11/107) in the United Kingdom, and 10.3% (11/107) in South Korea (Figure 3). Of note, only one study was conducted in India, one in China, and one in Africa.

RCTs (46.7%, 50/107) that assessed the differences between mAdherence tools or between an mAdherence tool and standard care were the most common study design. DM (62.6%, 67/107) interventions were the most common, followed by CVD (25.2%, 27/107) and CLD (17.8%, 19/107) interventions (Table 1). Six studies targeted both DM and CVDs and were included in both categories. Study durations ranged from just a few hours to 18 months, with an average duration of around 6 months. Sample sizes also varied widely, ranging from 4 to 710 participants.

**Table 1:** Study designs by chronic disease (n=107).

Study design	Diabetes mellitus,n (%)	Cardiovascular disease,n (%)	Chronic lung diseases,n (%)
**Randomized controlled trial**	29^a^ (27.1%)	17^a^ (15.9%)	9 (8.4%)
**Descriptive/feasibility**	16^b^ (15.0%)	2^b^ (1.9%)	9 (8.4%)
**Longitudinal/Pre- and Post-**	7 (6.5%)	6 (5.6%)	0 (0%)
**Quasi-experimental**	8 (7.5%)	1 (0.9%)	1 (0.9%)
**Crossover**	7 (6.5%)	0 (0%)	0 (0%)
**Retrospective**	0 (0%)	1 (0.9%)	0 (0%)
**Total**	67 (62.6%)	27 (25.2%)	19 (17.8%)

^a^Five articles included here considered both cardiovascular disease and diabetes mellitus.

^b^One article included here considered both cardiovascular disease and diabetes mellitus.

### mAdherence Users

Several of the studies focused on the use of mAdherence tools by vulnerable, hard-to-reach, or otherwise high-risk patient populations. This included elderly patients, members of minority ethnic and racial groups, and low-income adults. The characteristics of the target user group was often the impetus for the development of the mAdherence tool. For example, researchers noted that travel to a health care provider’s office can be difficult for older patients, and that mAdherence tools could lessen that burden [17]. Some studies also considered specific design considerations, such as larger device screens, that could make mAdherence tools easier to use by older adults [18]. In general, mAdherence tools targeting low-income, elderly, and minority groups were found to be usable with high satisfaction ratings [17-21]. In most of these interventions, mobile phones or other devices were either provided to users or considered a requirement for study participation. In a study that did not provide a mobile device to participants, access to mobile phones was noted to be a significant barrier [22].

In a study addressing the lack of knowledge in designing mHealth interventions for low-income and racial or ethnic minorities with DM, the authors noted that very little is known about decisions made in the mHealth design process for these patient populations [23]. An iterative design process involving systems and content development and multiple stages of user experience testing was recommended as a template for future mAdherence tools aimed at similar patient populations [23]. Ultimately, it appears that diverse individuals can use mAdherence tools as long as the tools are tailored to the needs of the population and sufficient training and support are provided [18,23].

### Mobile Tools Used in mAdherence

**Figure 4:**
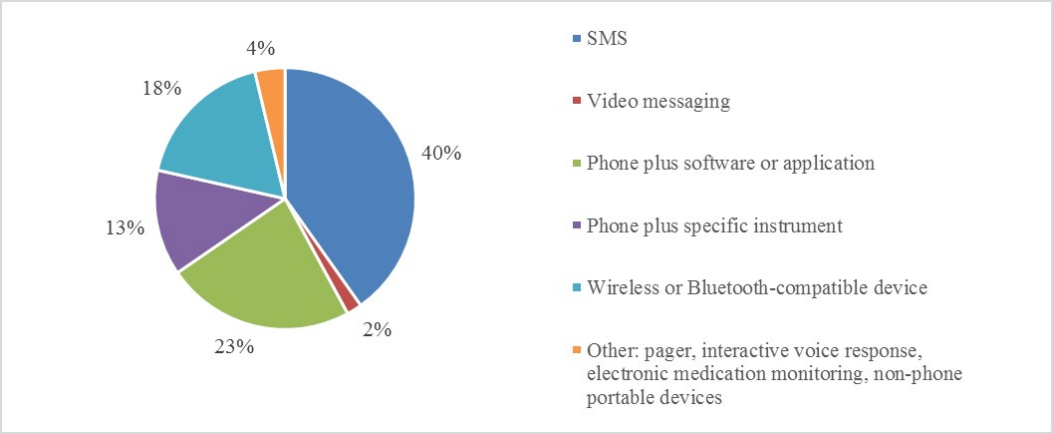
Types of mobile tools used in mAdherence.

For the purposes of this analysis, we classified mAdherence tools and platforms into four main categories: SMS; phone plus software or application; phone plus specific instrument (medical device connected to phone via a cord); or phone plus wireless or Bluetooth-compatible device (Figure 4). SMS interventions require the least sophisticated hardware and can be used to transmit simple information from patients on their personal phones. Specialized software or applications including patient portals, management systems, and other complex communication platforms require only a commercially available smartphone. Here, patients generally need to manually input information. Wireless or Bluetooth-compatible refers to medical devices used by patients that transmit information wirelessly to mobile phones and computers for viewing by both patients and health care providers. Phones plus a specific instrument require additional medical hardware usually not available on a commercial smartphone.

SMS (40.2%, 43/107) was the most commonly used tool and the primary platform. SMS facilitated patient-provider communication, medication reminders, and data collection and exchange on disease-specific measurements, as well as delivered patient education and motivation [24-26]. It is important to note that while SMS was often a feature of more complex patient-provider communication platforms, the 40.2% (43/107) of studies here used SMS exclusively. The next most common mAdherence tool was specialized software or a smartphone app, used in 23.4% (25/107) of studies. Use of specialized software applications was high among patients with DM. For example, mAdherence software could be installed on the patient’s mobile phone to help remember to check symptoms, maintain a food diary, or connect patients to DM educators in real time.

A wireless or Bluetooth-compatible device was used in 17.8% (19/107) of studies and a specific instrument connected to a phone, such as a blood glucose (BG) meter, was used in 13.1% (14/107) of studies. These mAdherence programs focused mainly on a combination of devices such as an electrocardiogram, BP monitor, and weighing machine with a wireless or Bluetooth interface, thus facilitating transfer of data automatically without requiring the patient to manually submit the data [27]. Data could then be reviewed by the health care provider and used to recommend an appropriate course of action. In some systems, automated criteria-based alerts were created, initiating an immediate response from the provider when measurements fell outside the target range [27,28]. CVD mAdherence programs also allowed for supervised cardiac rehabilitation by a remote monitoring system for those unable to access hospital-based programs [19].

### Study Outcomes and Indicators

Multiple outcome measures were used to evaluate mAdherence depending on stated study objectives. For the purposes of this analysis, the outcomes are organized into three categories: usability, feasibility, and acceptability of the mAdherence tool; effect of the mAdherence intervention on adherence to chronic disease management; and effect of the mAdherence intervention on disease-specific outcomes. In all, 62 studies (57.9%, 62/107) assessed usability, feasibility, and acceptability using qualitative methods and compiled usage data. These data ranged from patient satisfaction to cost-effectiveness estimations as well as timing and frequency of engagement with mobile tools and platforms. A total of 73 studies (68.2%, 73/107) evaluated the effect of an mAdherence intervention on adherence to chronic disease management, including medication adherence, engagement in healthy behaviors, frequency of symptom monitoring, and gains in knowledge and perceived self-efficacy. A total of 60 studies (56.1%, 60/107) assessed the effect of mAdherence on disease-specific clinical outcomes. Common clinical outcomes for DM included HbA1c, frequency of hypoglycemic events, and changes in insulin dosage. CVD measurements included changes in BP, lipid profile, and other biomarkers, as well as CVD risk profile. Examples of improved management of CLDs included indications of lung function, use of nebulizers, and exercise tests.

### Usability, Feasibility, and Acceptability

A total of 57.9% (62/107) of studies assessed usability, feasibility, and acceptability or patient preferences for mAdherence interventions. In general, the studies found mAdherence tools and platforms to be usable, feasible, acceptable, and appreciated among users. The majority of studies focused on the patient as the end-user of mAdherence, though some also looked at acceptability from the provider perspective. Features of mAdherence tools such as automated reminders, text messages with educational and motivational content, healthy living challenges, and wireless transmission of data contributed to increased self-care awareness and knowledge about chronic diseases [29-33]. mAdherence tools facilitated better management and improved patient confidence to monitor chronic diseases, making the patients feel in control and strengthening coping mechanisms [34]. Patients expressed feeling reassured, with decreased anxiety, knowing that their health symptoms were regularly monitored [28]. Often, having the mAdherence system as an interface between the patient and the provider was perceived as less burdensome and judgmental compared to face-to-face contact, particularly in situations in which the patients were not fully adherent to the recommended treatment [34].

The feasibility and acceptability of mAdherence tools were evaluated across diverse patient populations, including low-income, bilingual populations, and otherwise difficult-to-reach patients. The majority of participants included in these studies reported good comprehension and satisfaction [20,21,35-37]. For example, both adolescent patients with DM and their parents perceived that using an mAdherence system increased the adolescent’s independence and confidence in disease management [25,37,38]. Adolescent patients gave high ratings regarding the usefulness and feasibility of mAdherence systems to help them remember to take their medications and be attentive to their symptoms [21,25,26,36,39]. Parents of adolescent patients appreciated the decreased burden of reminding their children to perform required testing and self-care and noted decreased parent-adolescent conflict [25]. Among elderly populations, mAdherence was accepted and considered especially useful among older patients living alone and/or with memory issues [17]. One study found that the use of the mHealth DM tool studied was conditioned by gender [40]. Men and women were motivated by different priorities in their dietary self-efficacy and wanted different information, and the authors urge that gender be taken into account for future mHealth interventions. Physician providers also favored an mAdherence system that provided patient data and supported clinical decision-making [28].

Though mAdherence tools were generally accepted, patients and providers documented a number of negative elements and perceptions. Patients’ primary concerns included dependence on professional supervision, unnecessary medicalization, and undue anxiety if technology failed [30,34]. Difficulty in understanding and using the technology were reported, including technical issues such as too many menus to navigate and small buttons on the mobile phone [31]. Some patients who had not used smartphones before found them frustrating to use [32]. Among providers, concerns included the amount of time and effort required to review data and respond in time [41]. While studies confirmed that mAdherence tools are feasible in low-income populations, cost remains a barrier to more widespread use [22]. Factors such as the cost of implementing the system, increased clinical workload and workflow, maintaining up-to-date records, and concerns about being supervised and depending too much on technology were some of the main concerns regarding implementation of mAdherence interventions [28,30,34].

### Impact on Adherence

Only the subset of studies that employed a randomized comparison between two groups was included in this analysis. Descriptive studies and studies that did not involve a comparison group were excluded, as their diverse designs and methods prevented meaningful comparisons. Of the 27 RCTs that measured the effect of mAdherence on adherence behaviors, a statistically significant change or difference between groups (*P*<.05 to *P*<.001) was observed in 15 studies (56%) (Table 2). Multimedia Appendix 1 provides an overview of the methods and outcomes of these studies [18,33,35,37,42-64].

Two studies (4%, 2/27) found mixed results and 10 (37%, 10/27) showed no difference. Use of daily SMS reminders for medication intake with and without real-time medication monitoring showed significant improvements in patient adherence rates [42-46]. Text messaging tailored to counteract negative beliefs about asthma and education to overcome external barriers were associated with improved adherence to medication [43,47]. One study demonstrated the dual benefits of both better access to patient data and mobile coaching [65]. For adolescent patients with DM, employing automated, scheduled SMS programs providing motivational support was associated with improved adherence, understanding, and attention to DM care [45,48]. SMS notifications were particularly effective in increasing adherence to medication after a cardiac event [49,50]. Notably in one study, the use of an electronic blister pack with SMS communication significantly improved adherence to DM medication only and not to other types of medication [51].

**Table 2:** Effectiveness of mAdherence on adherence outcomes.

	Significant effect,n (%)	No significant effect,n (%)	Mixed results,n (%)	Total
**Diabetes mellitus**	7^a^ (50%)	5 (36%)	2 (14%)	14^a^
**Cardiovascular disease**	5^a^ (83%)	1 (17%)	0 (0%)	6^a^
**Chronic lung diseases**	4 (50%)	4 (50%)	0 (0%)	8
**Total adherence outcome studies**	15 (56%)	10 (37%)	2 (7%)	27

^a^One article is included here in both cardiovascular disease and diabetes mellitus.

### Impact on Clinical Outcomes

In all, 41 studies (38.3%, 41/107) evaluated the impact of mAdherence tools on clinical outcomes (Table 3). Of the RCTs that measured the effect of mAdherence on disease-specific clinical outcomes, significant differences between groups (*P*<.05 to *P*<.001) were reported in 16 studies (39%, 16/41). No significant differences were found in 14 studies (34%, 14/41), and mixed results were observed in 11 (27%). Multimedia Appendix 2 provides an overview of the methods and outcomes of these studies [18,23,27,30,33,35,39,42,43,45,46,48,49,52-55,57-61,65-83].

A total of 26 of the RCT interventions were related to improving DM management and care. Significant improvements in DM-specific clinical outcomes such as BG, HbA1c, and two-hour postprandial BG were reported in 11 studies (42%, 11/26). Both adolescents and elderly patients receiving messages with tailored instructions on DM care experienced statistically significant improvements in their HbA1c levels [18,31,45,46,66,67,84]. A total of 13 studies evaluated mAdherence tools for CVDs. Significant improvements in clinical outcomes such as BP, weight, and lipid profile were reported in 7 studies (54%, 7/13). In one study, SMS enabled interactive monitoring so that the provider could set reminders for patients with HTN, collect data, and schedule visits for treatment adjustments [68]. This resulted in 77% of patients achieving goal BP levels. Pairing data transfer with a criteria-based alarm system that alerted and initiated contact from the physician was associated with a significant decrease in systolic BP [27]. Significant reduction in BP was also observed among HTN patients using an electronic salt sensor and mobile phone [69]. Patients with risk factors for coronary artery disease showed significant improvement after using an mHealth system consisting of an automatic sphygmomanometer, BG and lipid meter, and mobile phone [70]. Four interventions were designed to improve outcomes for patients with both DM and CVD and half these studies showed significant improvements in clinical outcomes, including HbA1c and BP control [71-74]. Mixed results in CLD clinical outcomes, mainly lung function parameters, were reported in 3 (50%) of 6 RCTs that evaluated mAdherence for CLD, and the other 3 RCTs found non-significant results. SMS interventions improved cough symptoms and sleep quality [52]. Among COPD patients, use of mobile phones installed with music software to record respiratory symptoms during their exercise training showed a significant increase in the walking distance of the incremental shuttle walk test compared to the control group [53].

**Table 3:** Effectiveness of mAdherence on clinical outcomes (n=41).

	Significant effect,n (%)	No significant effect,n (%)	Mixed results,n (%)	Total
**Diabetes mellitus**	11^a^ (42%)	9 (35%)	6^a^ (23%)	26^b^
**Cardiovascular disease**	7^a^ (54%)	2 (15%)	4^a^ (31%)	13^b^
**Chronic lung diseases**	0 (0%)	3 (50%)	3 (50%)	6
**Total clinical outcome studies**	16 (39%)	14 (34%)	11 (27%)	41

^a^Two articles are included here in both cardiovascular disease and diabetes mellitus.

^b^Four articles are included here in both cardiovascular disease and diabetes mellitus.

## Discussion

### Principal Findings

The evidence presented here indicates that while the potential of mAdherence tools is high, their implementation and execution is mixed. In all, 50 of the studies employed RCT methodology, and of those, just more than half demonstrated significant effects on adherence (56%) and less than half (40%) on clinical outcomes. SMS is the mHealth tool most widely, frequently, and successfully used to facilitate adherence to chronic disease management. Able to be used by those with little technology experience or familiarity, SMS can be made available relatively inexpensively on any mobile phone, and can be automated, personalized, and easily integrated into existing health systems. However, it is highly operator dependent, relying on the active engagement of patients and providers to monitor symptoms and exchange information, and there is clearly room for improvement. The freedom and portability of mobile devices combined with the advanced capacity to facilitate two-way communication and collect and analyze data for a real-time response offer enormous potential to patients and providers. The potential complexity of today’s mAdherence tools and the mixed evidence in support of their effectiveness call for a renewed focus on understanding the connection between patient experience, adherence, and health outcomes.

More than half of the studies employed qualitative methods that yielded rich data that can be used to better understand how and why mAdherence tools impact adherence behaviors and clinical outcomes. User feedback can inform hypotheses that can then be tested. There is a growing understanding of barriers to adherence and ways to overcome them. mAdherence tools should be conceived, designed, developed, and evaluated with these barriers in mind. Research that seeks to understand how and why mAdherence works will deliver on the broader promise of mHealth. Future mHealth tools will be able to draw on the knowledge generated when discrete hypotheses around the relative importance of, for example, patient-provider communication, optimal user-interfaces, or targeted motivational messages are tested. This could lead to better mAdherence tools that deliver improved health outcomes.

This review found that the usability, feasibility, and acceptability of mHealth tools for chronic disease management adherence were generally high among both patients and providers. Innovative mAherence tools could unintentionally increase health disparities due to unequal access to technology. Vulnerable, hard-to-reach, or otherwise high-risk patient populations were the target audiences for several mAdherence interventions. There is a clear recognition that mHealth tools have the potential to impact patients who are less inclined to engage traditional health services. mAdherence offers a way to address barriers to care and to reduce health disparities. There is also some recognition that unequal access to, use of, and knowledge of information and communication technology can influence the uptake and use of mHealth tools. These inequalities and the needs of the target user group should be taken into consideration early in the design and development of the mAdherence tool. However, none of the studies included in this review addressed systematic differences in usability between diverse patient groups. Future research can be designed to better understand these differences and to encourage the development of mAdherence tools that address the needs of diverse patient groups.

Of note, few studies take seriously the issue of cost. In many of the small pilot studies, expensive devices or vouchers were given to study participants. When implemented at scale, interventions that use patients’ existing mobile devices rather than relying on gifted devices will go further toward explaining feasibility and improving adherence. Though currently concentrated in the developed world, pockets of mAdherence innovations are expanding around the globe. As developing countries work to address the burden of chronic disease, they may look to the potential of mHealth to lessen that burden. Part of that potential is to reduce costs and expand outreach. More mAdherence studies from resource-limited settings, especially in Africa and Asia, are needed. Rigorous cost-effectiveness analyses will be necessary to demonstrate not only the health impact but also the value of investing in these innovations now.

Besides cost, language, and literacy barriers, availability and connectivity issues are also potential barriers to consider. Perhaps most critically, if adherence to chronic disease management is not encouraged and actively practiced, it is very unlikely that mAdherence will be successful. mHealth tools are communication platforms and delivery mechanisms, not solutions in and of themselves. mAdherence will only work where there is already a functioning adherence program in place. Our review demonstrates that mAdherence can play a key role in translating mHealth technologies into better health outcomes. This role is becoming more explicit as mHealth research moves forward.

### Limitations

There are limitations to this systematic review. It is not a meta-analysis, and we did not weigh the quality of evidence or study design against reported results. We also did not include non-English literature, and some of the studies included as few as four participants. The diversity of study objectives, designs, and outcomes made clear comparisons difficult and the quality of evidence was variable.

Our review expands the current evidence base regarding the impact of mHealth on chronic disease management adherence by including common chronic diseases, extending the definition of mHealth beyond SMS to other types of mobile and wireless communication, and by assessing both self-management outcomes and the nascent literature regarding mHealth feasibility, usability, and acceptability.

### Conclusion

mAdherence is a potential high-impact tool to improve health outcomes among those living with chronic diseases through enhanced chronic disease management adherence. Further evaluation of mAdherence tools will be critical, especially research that informs how these tools overcome barriers to chronic disease management. More innovation, optimization, and high-quality research in mAdherence has the potential to transform the promise of mHealth technology into the reality of improved health care delivery and outcomes.

## Multimedia Appendix 1

Randomized controlled trials that measured impact of mHealth tools on patient adherence (n=27).

## Multimedia Appendix 2

Randomized controlled trials that measured impact of mHealth tools on patient clinical outcomes (n=41).

## Abbreviations

2Hpg: two-hour plasma glucose
AACM: active ambulatory care management
ACQ: asthma control questionnaire
ACS: acute coronary syndrome
ACT: asthma control test
AQLQ: asthma-specific quality-of-life questionnaire
baPWV: brachial-ankle pulse wave velocity
BG: blood glucose
BMI: body mass index
BNP: brain natriuretic peptide
BP: blood pressure
CARDS: Computerized Automated Reminder Diabetes System
CDSS: clinical decision support system
CGM: continuous glucose monitoring
CHD: coronary heart disease
CI: confidence interval
CIT: conventional insulin therapy
CLD: chronic lung disease
COPD: chronic obstructive pulmonary disease
CPDS: coach PCP portal with decision support
CPP: coach PCP portal
CRF: cardiovascular risk factors
CVD: cardiovascular disease
DBP: diastolic blood pressure
DID: diabetes interactive diary
DM: diabetes mellitus
ECG: electrocardiogram/electrocardiography
eNO: exhaled nitric oxide
FEV1: forced expiratory volume in one second
HbA1c: hemoglobin A1c
HCP: health care provider
HDL: high-density lipoprotein
HIV: human immunodeficiency virus
HRQL: health-related quality of life
HTN: hypertension
KASE-AQ: knowledge, attitude, and self-efficacy asthma questionnaire
LDL: low-density lipoprotein
LVEF: left ventricular ejection fraction
MEMS: Medication Event Monitoring System
MeSH: Medical Subject Headings
NICHE: Novel, Interactive Cell-phone Technology for Health Enhancement
PCA: perceived control of asthma
PCAQ-6: six-item PCA questionnaire
PCP: primary care provider
PDA: personal digital assistant
PEF: peak expiratory flow
RCT: randomized controlled trial
RR: risk ratio
RTM: remote telemedical management
RTMM: real-time medication monitoring
SBP: systolic blood pressure
SD: standard deviation
SMBG: self-monitoring of blood glucose
SMS: short message service
TACM: traditional ambulatory care management
TExT-MED: text message-based mHealth program to improve diabetes management
TM: telemonitoring
UCDC: ubiquitous chronic disease care
WAP: wireless application protocol
