# Supplementary material for: Impact of mHealth Chronic Disease Management on Treatment Adherence and Patient Outcomes: A Systematic Review
Source: J Med Internet Res. 2015 Feb 24;17(2):e52. doi: 10.2196/jmir.3951 (PMC4376208; doi:10.2196/jmir.3951)
Supplement: Supplementary file 1 [file jmir_v17i2e52_app1.pdf]

Reference	mHealth tool used	Study design	Control group	Intervention group	Outcome measures	Significance	Results
**Diabetes mellitus (n=13)**							
Arora et al. 2014 [35]	SMS<br>TExT-MED	• Intervention group: (n=64)<br>• Control group: (n=64)<br>• Study period: 6 months<br>• Adults with poorly controlled diabetes	The control group received usual care.	The TExT-MED group received 2 daily text messages for 6months in English or Spanish.	• Medication adherence<br>• self-efficacy<br>• performance of self-care tasks<br>• quality of life<br>• diabetes-specific knowledge	Mixed results.	• Change observed in self-reported medication adherence improved from 4.5 to 5.4 in the TExT-MED group compared with a net decrease of –0.1 in the controls (D1.1 [95% CI 0.1 to 2.1]).<br>• Changes in other outcomes were not statistically significant

Reference	mHealth tool used	Study design	Control group	Intervention group	Outcome measures	Significance	Results
Benhamou et al. 2007 [54]	Mobile phone capable of infrared data transmission and a PDA preloaded with gluconet® software.	• Patients with longstanding inadequately controlled diabetes (24 ± 13 years).<br>• Intervention group (n = 15).<br>• Control group (n = 15).<br>• Crossover after 6 months.<br>• Study period: 12 months.	Patients were requested to download their BG values at weekly intervals over 1 year, and to download the quality-of-life questionnaire every 3 months, within 1 week before or after clinic visits.	The gluconet® module was used to create and manage patient files, to display BG values and comments on graphic interface, and to send therapeutic advice via SMS. SMS transmission was unidirectional from investigator to patient without reply feature.	Adherence was determined from the server as the average number of BG tests performed by the patients (SMBG) during the week preceding each visit.	No significant difference.	• In the SMS to no-SMS group, initial SMBG value was 4.79 ± 1.74 tests per day and final value 4.63 ± 1.21 at 6 months.<br>• In the no-SMS to SMS group, initial value was 4.85 ± 1.34 and final value 4.74 ± 1.05 at 6 months.

Reference	mHealth tool used	Study design	Control group	Intervention group	Outcome measures	Significance	Results
Cho et al. 2009 [55]	Mobile phone containing a device to measure capillary BG onsite and transmit data to a web server.	• Control group (n = 37).<br>• Intervention group (n = 38).<br>• Study period: 3 months.<br>• Average age: 45 years control group; 51 years intervention group.	Internet group: Participants were taught about accessing and using the specialized, web-based diabetes patient management system and how to communicate with a management team through their individualized, web-based charts on the website at least once every other week. They entered their SMBG data on their web chart.	Mobile phone group: Participants were also taught how to perform their SMBG measurements and were given information about diet, physical exercise, and managing hypoglycemia. Data from patients in this group were automatically transmitted to a web server.	Adherence to treatment recommendations.	No significant difference.	Similar number of subjects replied that they accomplished the tasks as recommended by the doctor either online or by phone (77% vs. 83%, $P = 0.999$).

Reference	mHealth tool used	Study design	Control group	Intervention group	Outcome measures	Significance	Results
De Salvo et al. 2014 [56]	Dexcom® (San Diego, CA) G4™ PLATINUM CGM system, and Dexcom® G4™ PLATINUM CGM system communicating with the Diabetes Assistant.	• n = 20.<br>• Three camp sessions lasting 5–6 days each.	Ten campers were randomized to usual wear of a Dexcom® system.	Ten were randomized to remote monitoring with the Dexcom® system with the Diabetes Assistant, a mobile phone platform, to allow wireless transmission of CGM values.	• Response to alarm of hypoglycemic event.<br>• Duration of hypoglycemic event.	Significant difference.	• With remote monitoring, 100% of alarms were responded to; whereas, without remote monitoring, only 54% of alarms were responded to.<br>• The median duration of hypoglycemic events <70 mg/dL was 35 minutes without remote monitoring and 30 minutes with remote monitoring ($P = 0.078$).<br>• Remote monitoring significantly decreased prolonged hypoglycemic events, eliminating all events <50 mg/dL lasting longer than 30 minutes as well as all events <70 mg/dL lasting more than 2 hours.

Reference	mHealth tool used	Study design	Control group	Intervention group	Outcome measures	Significance	Results
Franklin et al. 2006 [45]	Sweet Talk, a text messaging support system.	• Control group (n = 28).<br>• Intervention group: CIT and Sweet Talk (n = 33), or intensive insulin therapy and Sweet Talk (n = 31).<br>• Study period: 12 months.<br>• Patients ages 8-18 years with type 1 diabetes.	CIT delivered by multidisciplinary team including clinic visits every 3-4 months and access to an emergency hotline.	Received CIT and Sweet Talk. Goal-setting at clinic visits was reinforced by daily text messages from the Sweet Talk software system, containing goal-specific prompts and messages tailored to each patient's age, sex, and insulin regimen, in addition to CIT.	Behavioral change measured by:<br>• Self-efficacy for diabetes score (alpha-reliability 0.9).<br>• Diabetes knowledge score (alpha-reliability 0.83).<br>• The diabetes social support interview (alpha-reliability 0.72–0.97).	Significant difference.	• Improved self-reported adherence score (CIT alone 70.4 ± 20.0; CIT with Sweet Talk 77.2 ± 16.1; 95% CI +0.4, +17.4; *P* < 0.05).<br>• Improved diabetes self-efficacy (conventional therapy 56.0 ± 13.7; conventional therapy plus Sweet Talk 62.1 ± 6.6; 95% CI +2.6, +7.5; *P* = 0.003) and self-reported adherence (conventional therapy 70.4 ± 20.0; conventional therapy plus Sweet Talk 77.2 ± 16.1; 95% CI +0.4, +17.4; *P* = 0.042).<br>• 82% of patients felt that Sweet Talk had improved their diabetes self-management.

Reference	mHealth tool used	Study design	Control group	Intervention group	Outcome measures	Significance	Results
Hanauer et al. 2009 [46]	CARDS, including a web-based module and a messaging/ reminder module.	• Email reminder group (n = 18).<br>• Mobile phone reminder group (via SMS) (n = 22).<br>• Study period: 3 months.<br>• Average age: 18 years.	Email group: CARDS sent reminder to participants via email to measure and submit BG measurement. Positive feedback was sent on submission, with warning for appropriate action if submitted value out of normal range. Repeat reminders sent at regular intervals if no response was received after first reminder.	Mobile phone group: CARDS sent reminder to participants via text messaging to measure and submit BG measurement. Positive feedback was sent on submission, with warning for appropriate action if submitted value out of normal range. Repeat reminders sent at regular intervals if no response was received after first reminder.	Adherence to submission of BG measurements based on number of measurements submitted by each user after receiving a reminder from CARDS.	Significant difference in first month of study only.	• 18 in the mobile phone group and 11 in the email group used the system.<br>• Compared to the email group, users in the mobile phone group received more reminders (180.4 vs. 106.6 per user) and responded with BG results significantly more often (30.0 vs. 6.9 per user, *P* = 0.04).<br>• During the first month, mobile phone users submitted twice as many BG measurements as email users (27.2 vs. 13.8 per user).

Reference	mHealth tool used	Study design	Control group	Intervention group	Outcome measures	Significance	Results
Kim et al. 2007 [57]	Mobile phone with glucometer and pedometer device.	• Intervention group (n = 35).<br>• Control group (n = 36).<br>• Study period: 12 weeks.<br>• Type 2 diabetes patients, mean age 48.1 years.	Patients received glucometers and usual outpatient management from physicians.	Patients received an Internet-based diabetes management system using SMS. Data on BG, diet, and exercise were automatically uploaded via glucometer and pedometer.	Adherence to measuring BG, diet-related data input, and pedometer data transfer.	Significant difference.	• Intervention group checked BG more frequently than control group (167.1 ± 88.2 vs. 44.6 ± 24.4, $P < 0.001$) over 3 months.<br>• Diet and exercise data transfer in intervention group was 12.07 ± 3.0 and 56 ± 13.7 respectively.

Reference	mHealth tool used	Study design	Control group	Intervention group	Outcome measures	Significance	Results
Kirwan et al. 2013 [58]	Smartphone application (Glucose Buddy) with weekly text message feedback from a Certified Diabetes Educator.	• Glucose Buddy group (n = 25).<br>• Usual care group (n = 28).<br>• Study period: 9 months.<br>• Type 1 diabetes patients, mean age 35.2 years.	Patients were asked to continue with their usual care, which included a visit to their primary diabetes HCP every 3 months.	Patients were asked to continue with usual care and use Glucose Buddy.	• Selfefficacy.<br>• Selfcare activities.<br>• Quality of life.	No significant difference.	No significant change over time was found in either group in relation to self-efficacy, self-care activities, and quality of life.

Reference	mHealth tool used	Study design	Control group	Intervention group	Outcome measures	Significance	Results
Kumar et al. 2004 [48]	A handheld device fitted with a wireless modem and diabetes data management software, plus a wireless-enabled BG monitor.	• Control group (n = 21).<br>• Intervention group (n = 19).<br>• Study period: 4 weeks.<br>• Participant ages: 8-18 years.	Participants received a handheld PDA fitted with a wireless modem and diabetes data management software, plus a wireless-enabled BG monitor.	Received additional software called the DiaBetNet™ (Dimagi, New Delhi, India) on their PDA. The game prompted participants to predict their upcoming BG values, insulin doses, and carbohydrate intake.	• Participation rates.<br>• Number of transmissions between groups.<br>• BG monitoring.<br>• Child and parent self-reporting of diabetes knowledge and satisfaction.	Significant difference.	• 93% of participants transmitted their data wirelessly to the server. The intervention group transmitted significantly more BG values than the control group ($P < 0.001$).<br>• Youth in the intervention group displayed a significant increase in diabetes knowledge over the 4-week trial ($P < 0.005$).<br>• Parents and children found it easier to track BG but were less satisfied about being unable to share BG values with their physicians.

Reference	mHealth tool used	Study design	Control group	Intervention group	Outcome measures	Significance	Results
Lim et al. 2011 [18]	Individualized interactive u-healthcare service based on automated CDSS using SMS on mobile phones.	• Routine care group (n = 48).<br>• SMBG group (n = 47).<br>• u-healthcare group with wired mobile phone-connected glucometer (n = 49).<br>• Study period: 3 months.<br>• Diabetes patients, average age 67-68 years.	• Control group received no intervention; followed regular medical care.<br>• SMBG group measured their BG level at least 8 times a week.	• u-healthcare group used public switched telephone network-connected glucometers and measured BG at same frequency as SMBG group.<br>• Received SMS on mobile phones from the CDSS rule engine server based on patient's BG testing.<br>• Evaluation messages on BG levels and frequency of BG testing were also sent at weekly and monthly intervals.	Frequency of self-monitored BG.	Significant difference.	Frequency of SMBG was significantly increased in u-healthcare group compared to control group. 81.2% of patients in u-healthcare group achieved target frequency for testing, compared to 31.2% in control group ($P < 0.01$).

Reference	mHealth tool used	Study design	Control group	Intervention group	Outcome measures	Significance	Results
Shetty et al. 2011 [59]	SMS via mobile phones.	• Control group (n = 66).<br>• SMS group (n = 78).<br>• Study period: 12 months.<br>• Ages: 50 ± 8 years.	Received standard care, including prescription medications and advice on diet and lifestyle modification.	Received SMS once every 3 days as a reminder to strictly follow the regimen of dietary modification, physical activity, and medication schedules.	Adherence to medication prescriptions, diet, and physical activity compliance.	No significant difference.	• Adherence rate to diet prescription did not change significantly in either group: SMS group (60.3% vs. 58.4%) and control group (54.5% vs. 52%) at baseline and post 12 months, respectively.<br>• Adherence to physical activity improved marginally, from 47% to 56%, but the change was statistically non-significant.
Vervloet et al. 2012 [44]	RTMM system using an electronic medication dispenser to monitor patients' medication and register the data.<br>RTMM use combined with SMS reminders when patients forgot to take their medication.	• SMS group (n = 56).<br>• Control group (n = 48).<br>• Study period: 6 months.<br>• Patients with type 2 diabetes, predominantly male, average age 55 years.	Patients used RTMM medication dispenser but did not receive SMS support if medication use was not registered within agreed time period.	Patients received their medications in the RTMM medication dispenser, and received an SMS reminder if medication intake was not registered within the agreed time period.	Adherence to diabetes medications, measured using three different measures:<br>• Number of days without dosing (the dispenser was not opened at all).<br>• Proportion of missed doses.<br>• Proportion of doses taken within agreed and predefined standardized time windows.	Mixed results.	• Groups did not differ significantly with respect to average number of days without dosing.<br>• SMS group patients missed 5% fewer doses than patients not receiving SMS reminders ($P = 0.065$).<br>• Patients who received SMS reminders took more doses within the agreed time period compared to control group ($P = 0.003$).

Reference	mHealth tool used	Study design	Control group	Intervention group	Outcome measures	Significance	Results
Zolfaghari et al. 2012 [60]	Regular SMS messages related to diet, exercise, diabetes medications, and BG monitoring delivered to patients' mobile phones.	• SMS group (n = 39).<br>• Telephone group (n = 41).<br>• Study period: 3 months.<br>• Patients with type 2 diabetes, average age 51-54 years.	Participants received counseling on nature of disease, risk factors, importance of BG monitoring, and reinforcement of diet, exercise, medications, etc., over a telephone call.	Patients received SMS messages with information on diet, exercise, medication intake, BG monitoring, and stress management over their mobile phones—around 6 messages per week.	Self-reported adherence was measured by self-care diabetes questionnaire, including information on medication, diet, and physical exercise adherence.	No significant difference.	There was no significant difference in diet, physical exercise, and medication intake adherence in either group.
**Cardiovascular diseases (n = 5)**							
Carrasco et al. 2008 [61]	WAP sessions and SMS services.	• Telemedicine group (n = 142).<br>• Control group (n = 143).<br>• Study period: 6 months<br>• Hypertensive patients with the average age 62.	Patients followed the same BP monitoring protocol as telemedicine group. Results were recorded on paper and patients interacted with their HCP only during scheduled visits to the office.	Patients sent self-measured BP, pulse rate, and weight weekly and responded to questionnaire during each WAP session. HCPs accessed the data and could send SMS messages to patients regarding any health-related issue.	Adherence to protocol for BP monitoring assessed in both groups to all required steps outlined for monitoring procedure.	No significant difference.	Both groups presented similar adherence at >90% level (25.2% vs. 26.1%), while adherence was better but not statistically significant in the telemedicine group for >75% (59.6% vs. 53.6%), >50% (84.8% vs. 73.3%), and >25% (92.4% vs. 75.4%).

Reference	mHealth tool used	Study design	Control group	Intervention group	Outcome measures	Significance	Results
Khonsari et al. 2013 [50]	SMS medication reminder.	• Usual care (n = 31).<br>• Intervention (n = 31).<br>• Study period: 8 weeks.<br>• Patients with ACS, average age 58-59 years.	Patients with ACS received only usual care for 8 weeks after discharge.	Patients with ACS received automated SMS reminders before every intake of cardiac medications for 8 weeks after discharge.	Adherence to cardiac medications.	Significant difference.	• Higher medication adherence level in the intervention group than the usual care group ($\chi2$ (2) = 18.614, *P* < 0.001).<br>• Risk of being low adherent among the control group was 4.09 times greater than the intervention group (relative risk = 4.09, 95% CI 1.82–9.18).
Park et al. 2014a [62]	SMS medication reminder and electronic medication monitoring device.	• SMS education (n = 30).<br>• No SMS (n = 30).<br>• SMS reminders + SMS education (n = 30).<br>• Study period: 30 days.<br>• Patients with CHD, 76% male, average age 59.2 years.	Patients with CHD who received neither text messages regarding medication reminders nor educational messages.	• Patients with CHD who received text messages regarding educational messages only.<br>• Patients with CHD who received text messages regarding medication reminders and educational messages.	Medication adherence with MEMS and self-reported adherence (Morisky Medication Adherence Scale).	Significant difference.	• Total scores for medication self-efficacy improved over 30 days, there was no significant difference in this improvement as a function of the different treatment groups (*P*=0.64).<br>• Controlling for other variables in the model (age, education, depression, and social support), less depression (*P* =0.004) and higher social support (*P*=0.02) positively predicted higher medication adherence.

Reference	mHealth tool used	Study design	Control group	Intervention group	Outcome measures	Significance	Results
Park et al. 2014b [63]	SMS medication reminder and electronic medication monitoring device.	• SMS education (n = 30).<br>• No SMS (n = 30).<br>• SMS reminders + SMS education (n = 30).<br>• Study period: 30 days.<br>• Patients with CHD, 76% male, average age 59.2 years.	Patients with CHD who received neither text messages regarding medication reminders nor educational messages.	• Patients with CHD who received text messages regarding educational messages only.<br>• Patients with CHD who received text messages regarding medication reminders and educational messages.	Medication adherence with MEMS and self-reported adherence (Morisky Medication Adherence Scale).	Significant difference.	• Patients who received SMS for antiplatelet had a higher percentage of correct doses taken (P = 0.02), percentage number of doses taken (P = 0.01), and percentage of prescribed doses taken on schedule (P = 0.01).<br>• Self-reported adherence revealed no significant differences among groups.
Quilici et al. 2013 [49]	SMS medication reminder.	• SMS group (n = 250).<br>• Standard care group (n = 249).<br>• Study period: 1 month.<br>• ACS patients, average age 64 years.	After cardiac events, patients were discharged with a prescription of aspirin 75 mg and clopidogrel, and were provided with educational sessions highlighting the importance of adherence to recommendations.	After cardiac events, patients received standard care and received a daily personalized SMS.	One-month self-reported aspirin adherence and controlled aspirin adherence using platelet function testing.	Significant difference.	Controlled non-adherent patients assessed by platelet testing accounted for 11.2% of the standard care group vs. 5.2% in the SMS intervention group (OR [95% CI]: 0.43 [0.22–0.86]; P = 0.01).
**Chronic lung diseases (n = 8)**							

Reference	mHealth tool used	Study design	Control group	Intervention group	Outcome measures	Significance	Results
Liu et al. 2008 [63]	Software installed on mobile phone to support exercise and monitor adherence to the program by sending exercise data to website.	• Mobile phone group (n = 24).<br>• Control group (n = 24).<br>• Study period: 12 months.<br>• Patients with moderate to severe COPD, average age 71-73 years.	Patients followed the same exercise protocol at home as the intervention group, but without the mobile phone program.	Music software with an individualized tempo was installed on patients' mobile phones. Patients participated in endurance exercise training by walking at a speed following the music. The mobile phone recorded the duration of music played, equal to the duration of walking.	Adherence to and compliance with home-based training exercise program by monitoring the frequency of performance and the duration of the endurance walking program every week.	Significant difference.	Higher proportion of patients in the mobile phone group (92%) were adherent to the home-based exercise program compared to the control group (38%) ($P < 0.01$).

Reference	mHealth tool used	Study design	Control group	Intervention group	Outcome measures	Significance	Results
Lv et al. 2012 [43]	SMS with reminders and information on asthma management.	• SMS group (n = 30).<br>• Traditional group (n = 27).<br>• Control group (n = 14).<br>• Study period: 12 weeks.<br>• Asthma outpatients.	• Control group: Participants received verbal asthma education from physicians during clinic visit.<br>• Traditional group: In addition to above asthma education, patients used a free PEF meter.	SMS group: In addition to receiving asthma education, patients received SMS reminders twice daily about how to manage asthma. Patients could also send SMS messages to clinic investigators if they had any questions and needed further guidance.	Follow-up adherence rate, medicine compliance rate, and emergency department visit data were collected at end of study period.	Significant difference.	• Participants in the SMS group showed a significantly higher follow-up adherence rate compared to traditional and control groups: 60% vs. 54% vs. 28%, respectively (P = 0.003).<br>• Medication compliance rate among those who completed the follow-up visit did not differ significantly between the three groups (P = 0.113), but rates were higher in the SMS group (80%) than the traditional (74%) and control (50%) groups.

Reference	mHealth tool used	Study design	Control group	Intervention group	Outcome measures	Significance	Results
Nguyen et al. 2009 [64]	SMS and mobile phone diaries.	• MOBILE-Coached (n = 9).<br>• MOBILE-Self-Monitored (n = 8).<br>• Study period: 6 months.<br>• Patients with COPD, average age 68 years.	Participants used their mobile phone to enter information about their symptoms and exercise. Participants did not receive any other prompting or personalized feedback and the symptom alert was disabled.	Participants submitted daily information about their symptoms and exercise. An alarm was set on the mobile phone calendar tool to remind participants to complete their entries. Ongoing reinforcement feedback was provided via SMS to the participant's mobile phone, by the nurse.	Participants completed incremental cycle and 6-minute walk tests, wore an activity monitor for 14 days, and reported their HRQL at baseline, 3, and 6 months.	No significant difference.	• There were no differences between groups over time in maximal workload, 6-minute walk distance, or HRQL.<br>• MOBILE-Self-Monitored increased total steps/day, whereas MOBILE-Coached logged fewer steps over 6 months (P = 0.04).

Reference	mHealth tool used	Study design	Control group	Intervention group	Outcome measures	Significance	Results
Ostojic et al. 2005 [52]	SMS.	• Study group (n = 8).<br>• Control group (n = 8).<br>• Study period: 16 weeks.<br>• Asthma patients, average age 24.6 years.	One-hour asthma education session with specialist and treatment. Control group noted PEF measurements, medication use, and symptoms in paper diary.	In addition to education, patients in the SMS group were instructed to send their PEF results daily via SMS and received weekly instructions from an asthma specialist on adjustments to therapy and follow-up based on PEF measurements sent.	Compliance with PEF data transmission using SMS was compared to compliance with PEF diary entries in control group.	No significant difference.	There was no significant difference in compliance with PEF measurement between the two groups (66% vs. 61%, P = 0.878) for the SMS group vs. control group.

Reference	mHealth tool used	Study design	Control group	Intervention group	Outcome measures	Significance	Results
Petrie et al. 2012 [47]	SMS.	• SMS group (n = 73).<br>• Control group (n = 74).<br>• Study period: 9 months.	Patients received normal care without any text message support.	Participants assigned to the text message group received tailored text messages for 18 weeks. Each of the texts was designed to counteract the specific illness and medication beliefs that had previously been found to be associated with non-adherence to preventer medication.	Adherence to asthma preventer inhalers was assessed at 6, 12, 18 weeks and at 6 and 9 months.	Significant difference.	• Average self-reported adherence over all time points was significantly higher in the intervention group (57.8%) compared to the control group (43.2%) (*P* = 0.003).<br>• The proportions with average adherence of 80% or more for the control group was 7 of 66 (10.6%) and for the intervention group 15 of 58 (25.9%). The difference between the two groups was 15.3% (*P* = 0.034).

Reference	mHealth tool used	Study design	Control group	Intervention group	Outcome measures	Significance	Results
Ryan et al. 2012 [33]	The t+ Asthma application for recording and transmission of symptoms, medication use, and peak flow.	• Mobile phone group (n = 145).<br>• Control group (n = 143).<br>• Study period: 6 months.<br>• Adolescents and adults with poorly controlled asthma.	Patients in the control (paper) group were asked to keep a paper diary, recording the same data as the intervention group (symptoms, medication use, and peak flow readings) twice daily.	Patients in the mobile phone group were provided with the t+ Asthma application, which enabled twice-daily recording and transmission of symptoms, medication use, and peak flow. Incursion into the red or amber zones triggered contact by an asthma nurse. Both patients and clinicians were able to access the patient data.	Asthma control was measured using the ACQ.	No significant difference.	• There was no significant difference in asthma control or self-efficacy between the two groups.<br>• ACQ: Mean change 0.75 in mobile phone group vs. 0.73 in paper group, mean difference in change −0.02 (95% CI −0.23 to 0.19).<br>• KASE-AQ score: Mean change −4.4 vs. −2.4, mean difference 2.0 (−0.3 to 4.2).
Seid et al. 2012 [37]	SMS.	• Intervention group (n = 12).<br>• Control group (n = 14).<br>• Study period: 3 months.<br>• At-risk adolescents with asthma, average age 15 years.	Received asthma education with no SMS.	Received asthma education, motivational interviewing, problem-solving skills training, and tailored SMS via mobile phone for 1 month.	• Manipulation check (i.e., did intervention increase motivation?).<br>• Participant activation, intention, and motivation.<br>• Adherence barriers.<br>• Asthma symptoms.<br>• HRQL.	No significant difference.	• Small differences between groups were noted; *P* values were not provided.<br>• The intervention group had more than 1 day fewer asthma symptoms in the previous 2 weeks vs. baseline and vs. the control group at both 1 and 3 months post baseline.

Reference	mHealth tool used	Study design	Control group	Intervention group	Outcome measures	Significance	Results
Strandbygaard et al. 2010 [42]	SMS.	• SMS group (n = 12).<br>• Control group (n = 14).<br>• Study period: 12 weeks.<br>• Patients with clinical history of asthma, ages 18-45 years.	Patients received prescription medications for last 4 weeks of study period, but did not receive any SMS reminders about medication intake.	Patients received the prescribed medications as well as daily SMS messages reminding them to take their asthma medication.	Medication adherence was measured based on medicine dose-count at the end of 4 weeks.	Significant difference.	• The adherence rate increased from 77.9% to 81.5% (*P* = 0.52) in SMS group, but significantly reduced in the control group, from 84.2% to 70.1% (*P* = 0.01).<br>• At the end of 4-week period, the difference in adherence rate between the two groups was 17.8% (95% CI 3.2–32.3%, *P* = 0.019).
**Diabetes mellitus + cardiovascular disease (n = 1)**							
Brath et al. 2013 [51]	Electronic medication blister pack plus mobile phone data gateway.	• Crossover study design, 20 weeks in each phase.<br>• Monitoring phase.<br>• Control phase.<br>• 53 patients with risk for cardiovascular disease, mean age 69 years.	Control phase characterized by standard medication blisters, routine care, and handwritten medication intake diaries.	Monitoring phase using the electronic medication blister pack.	Medication adherence.	Significant difference.	A difference (*P* = 0.04) between the monitoring and the control phase was observed for the diabetes medication only.
