# Supplementary material for: Impact of mHealth Chronic Disease Management on Treatment Adherence and Patient Outcomes: A Systematic Review
Source: J Med Internet Res. 2015 Feb 24;17(2):e52. doi: 10.2196/jmir.3951 (PMC4376208; doi:10.2196/jmir.3951)
Supplement: Supplementary file 2 [file jmir_v17i2e52_app2.pdf]

Reference	mHealth tool used	Study design	Control group	Intervention group	Outcome measures	Significance	Results
**Diabetes mellitus (n = 22)**							
Arora et al. 2014 [29]	TExT-MED.	• Intervention group (n = 64).<br>• Control group (n = 64).<br>• Study period: 6 months.<br>• Adults with poorly controlled diabetes.	Received usual diabetes care.	The TExT-MED group received 2 daily text messages for 6 months in English or Spanish.	HbA1c.	No significant difference.	HbA1c level decreased by 1.05% in the TExT-MED group compared with 0.60% in the control group (*P*=0.45; 95% CI 0.27–1.17) at 6 months.

Reference	mHealth tool used	Study design	Control group	Intervention group	Outcome measures	Significance	Results
Bell et al. 2012 [75]	Video messaging sent to mobile phones.	• Usual care (n = 33).<br>• Video messaging (n = 32).<br>• Study period: 6 months.<br>• Patients at endocrinology clinic at Walter Reed Health Care System with HbA1c >8%.	Received usual diabetes care.	Six nurse practitioners created 30- to 60-second videos covering self-care topics such as healthy eating, being active, and monitoring. Videos were sent to patients.	HbA1c.	Significant difference.	• Participants who received video messages had a larger rate of decline in HbA1c than people who received usual care (0.2% difference; $P = 0.002$ and $P = 0.004$ for the interaction between time and the group).<br>• HbA1c decline was greatest among participants who viewed >10 a month over 12-month period (0.6% difference; $P < 0.001$).

Reference	mHealth tool used	Study design	Control group	Intervention group	Outcome measures	Significance	Results
Benhamou et al. 2007 [64]	Mobile phone capable of infrared data transmission and a PDA preloaded with gluconet® software.	• Intervention group (n = 15).<br>• Control group (n = 15).<br>• Cross over after 6 months.<br>• Study period: 12 months.<br>• Patients with longstanding inadequately controlled diabetes.	Patients were requested to download their BG values at weekly intervals over 1 year, and to download the quality-of-life questionnaire every 3 months, within 1 week before or after clinic visit.	The gluconet® module was used to create and manage patient files, display BG values and comments on graphic interface, and send therapeutic advice via SMS. SMS transmission was unidirectional from investigator to patient without reply feature.	• HbA1c.<br>• Average glycemic value.	No significant difference.	A non-significant trend to reduction in HbA1c (–0.25 ± 0.94%; *P* = 0.097) and mean BG values (–9.2 ± 25 mg/dl; *P* = 0.064) during the 6-month SMS sequence was observed as compared with the no-SMS period.

Reference	mHealth tool used	Study design	Control group	Intervention group	Outcome measures	Significance	Results
Cho et al. 2009 [65]	Mobile phone containing a device to measure capillary BG onsite and transmit data to a web server.	• Control group (n = 37).<br>• Intervention group (n = 38).<br>• Study period: 3 months.<br>• Average age: 45 years control group; 51 years intervention group.	Internet group: Participants were taught about accessing and using the specialized, web-based diabetes patient management system and how to communicate with a management team on the website at least once every other week.	Mobile phone group: The patients were also taught how to perform their SMBG measurements and were given information about diet, physical exercise, and managing hypoglycemia. Data from patients in this group were automatically transmitted to a web server.	• HbA1c.<br>• Two-hour postprandial BG levels.<br>• Fasting BG.<br>• Total cholesterol.<br>• HDL and LDL cholesterol.<br>• Triglycerides.	No significant difference.	• HbA1c decreased in both groups, but the difference between groups was not different significantly (P = 0.27).<br>• Fasting plasma glucose levels were not significantly different between the two groups.<br>• No changes in total cholesterol, HDL cholesterol, triglyceride, and LDL cholesterol levels before and after intervention.

Reference	mHealth tool used	Study design	Control group	Intervention group	Outcome measures	Significance	Results
Faridi et al. 2008 [24]	NICHE. Wireless biometric devices to transmit data and send SMS messages to patients.	• Intervention group (n = 15).<br>• Control group (n = 15).<br>• Study period: 3 months.<br>• Patients with type 2 diabetes.<br>• Average age: 55-57 years.	Control subjects continued with standard diabetes self-management and tracked their step count using a pedometer.	Intervention patients received training in NICHE technology. Patients participated in a 3-month intervention and were required to test their BG once daily, wear their pedometers during the day, and upload data onto the NICHE server once daily. They received tailored messages via mobile phone based on the uploaded data.	• HbA1c.<br>• Trend analysis of glucometer readings between groups.<br>• BMI.	No significant difference.	Improvement in HbA1c levels was apparent but not statistically significant (−0.1, SD = 0.3%; *P* = 0.15) in the intervention group, compared with a mean deterioration in the control (0.3, SD = 1.0%; *P* = 0.38).

Reference	mHealth tool used	Study design	Control group	Intervention group	Outcome measures	Significance	Results
Franklin et al. 2006 [42]	Sweet Talk, a text messaging support system.	• Control group (n = 28).<br>• Intervention group: CIT and Sweet Talk (n = 33), or intensive insulin therapy and Sweet Talk (n = 31).<br>• Study period: 12 months.<br>• Patients ages 8-18 years with type 1 diabetes.	CIT delivered by multidisciplinary team including clinic visits every 3-4 months and access to an emergency hotline.	Received CIT and Sweet Talk. Goal-setting at clinic visits was reinforced by daily text messages from the Sweet Talk software system, containing goal-specific prompts and messages tailored to each patient's age, sex, and insulin regimen, in addition to CIT.	HbA1c.	Significant difference.	HbA1c did not change in patients on conventional therapy without or with Sweet Talk but improved in patients randomized to intensive therapy and Sweet Talk (9.2 ± 2.2%; 95% CI −1.9, −0.5; *P* < 0.001).

Reference	mHealth tool used	Study design	Control group	Intervention group	Outcome measures	Significance	Results
Hanauer et al. 2009 [43]	CARDS, including a web-based module and a messaging/ reminder module.	• Email reminder group (n = 18).<br>• Mobile phone reminder group (via SMS) (n = 22).<br>• Study period: 3 months.<br>• Average age: 18 years.	Email group: CARDS sent reminder to participants via email to measure and submit BG measurement. Positive feedback was sent on submission, with warning for appropriate action if submitted value out of normal range. Repeat reminders sent at regular intervals if no response was received after first reminder.	Mobile phone group: CARDS sent reminder to participants via text messaging to measure and submit BG measurement. Positive feedback was sent on submission, with warning for appropriate action if submitted value out of normal range. Repeat reminders sent at regular intervals if no response was received after first reminder.	HbA1c.	No significant difference.	• No change in glycemic control observed in the 2 groups after intervention.<br>• At baseline, the mean HbA1c values were 8.6 +/– 0.9% and 8.9 +/– 1.7% in the email and mobile phone users, respectively.<br>• After the study, the mean values remained similar, at 8.8 +/– 0.9% for email users and 8.7 +/– 1.5% for mobile phone users.

Reference	mHealth tool used	Study design	Control group	Intervention group	Outcome measures	Significance	Results
Harno et al. 2006 [50]	Diabetes management system application with provision for diabetes team to transmit SMS messages to patients with mobile phones.	• Study group (n = 101).<br>• Usual care (n = 74).<br>• Study period: 12 months.<br>• Patients with types 1 and 2 diabetes.	Regular general practitioner visits about every 3 months.	Patients downloaded their measurements directly from the BG meter into the regional database using a modem. The self-management system allowed the diabetes team to transmit SMS messages to patients with mobile phones and Internet access. The care team was able to access the home diary with the patient's consent.	• HbA1c.<br>• Diastolic BP.<br>• Fasting BG.<br>• Serum total cholesterol.<br>• Serum LDL cholesterol.<br>• Serum triglycerides.	Significant difference.	• HbA1c was significantly lower in the study group than the control group.<br>• DBP, fasting BG, serum total cholesterol, serum LDL cholesterol, and serum triglycerides were significantly lower in the study group than the control group.

Reference	mHealth tool used	Study design	Control group	Intervention group	Outcome measures	Significance	Results
Istepenian et al. 2009 [57]	BG sensor that transmitted readings to a mobile phone via a Bluetooth wireless link.	• TM group (n = 72).<br>• Control group (n = 65).<br>• Study period: 9 months.<br>• Patients with diabetes, average age 61 years.	Patients in the control group did not use a mobile phone to transmit data and received their care from a diabetes center and local practitioners.	Patients in the TM arm were trained to measure BG. The monitor was adapted to transmit their recordings wirelessly by Bluetooth to a mobile phone according to a personalized monitoring schedule. The mobile phone alerted the patient when a measurement was due. Data were sent and reviewed by the research clinicians via a web-based application.	HbA1c.	No significant difference.	There were no differences in HbA1c between the TM and the control groups: 7.99% and 8.2%, respectively ($P = 0.17$).

Reference	mHealth tool used	Study design	Control group	Intervention group	Outcome measures	Significance	Results
Kim and Jeonge 2007 [52]	SMS messages from nurses and diabetes website.	• Intervention group (n = 25).<br>• Control group (n = 26).<br>• Study period: 6 months.<br>• Adult diabetes patients.	Met the endocrinology specialist once or twice during the 6 months.	Used website to send their SMBG levels and medication information. Patients were sent recommendations weekly, by mobile phone SMS or wired Internet.	• HbA1c.<br>• Fasting BG levels.<br>• Two-hour post-meal BG.	Mixed results.	• HbA1c decreased 1.15% points at 3 months and 1.05% points at 6 months compared with baseline in the intervention group ($P < 0.05$).<br>• Fasting BG did not differ significantly in either group.<br>• Patients in the intervention group had a decrease of 2 hours post-meal BG of 85.1 mg/dl at 3 months and 63.1 mg/dl at 6 months compared with baseline ($P < 0.05$).

Reference	mHealth tool used	Study design	Control group	Intervention group	Outcome measures	Significance	Results
Kim et al. 2007 [67]	Mobile phone with glucometer and pedometer device.	• Intervention group (n = 35).<br>• Control group (n = 36).<br>• Study period: 12 weeks.<br>• Type 2 diabetes patients, mean age 48.1 years.	Patients received glucometers and usual outpatient management from physicians.	Patients received an Internet-based diabetes management system using SMS. Data on BG, diet, and exercise were automatically uploaded via glucometer and pedometer.	• Anthropomorphic data.<br>• BP.<br>• Biochemical profiles.	Significant difference.	• Weight decreased from 66.6 to 64.7 kg in the intervention group (*P* = 0.037). No change was observed in the control group (69.7 to 69.2 kg; *P* = 0.117).<br>• Reduction in HbA1c was observed in the intervention group (from 8.06 to 7.34; *P* = 0.001), but not in the control group.<br>• Fasting and postprandial BG decreased in the intervention group from 159.4 to 132.3 mg/dl (*P* < 0.001), and in the control group from 233.4 to 180.9 mg/dl (*P* = 0.001).

Reference	mHealth tool used	Study design	Control group	Intervention group	Outcome measures	Significance	Results
Kirwan et al. 2013 [68]	Smartphone application (Glucose Buddy) with weekly text message feedback from a Certified Diabetes Educator.	• Glucose Buddy group (n = 25).<br>• Usual care group (n = 28).<br>• Study period: 9 months.<br>• Type 1 diabetes patients, mean age 35.2 years.	Patients were asked to continue with their usual care, which included a visit to their primary diabetes health care practitioner every 3 months.	Patients were asked to continue with usual care and use Glucose Buddy.	HbA1c.	Significant difference.	The intervention group had a significant decrease in HbA (mean −1.10; SD 0.74; *P* < 0.001) over the 9-month study, compared to the control group, which did not have a significant increase.

Reference	mHealth tool used	Study design	Control group	Intervention group	Outcome measures	Significance	Results
Kumar et al. 2004 [46]	A handheld device fitted with a wireless modem and diabetes data management software, plus a wireless-enabled BG monitor.	• Control group (n = 21).<br>• Intervention group (n = 19).<br>• Study period: 4 weeks.<br>• Ages: 8-18 years.	Participants received a handheld PDA fitted with a wireless modem and diabetes data management software, plus a wireless-enabled BG monitor.	Intervention group received additional software called the DiaBetNet™ (Dimagi, New Delhi, India) on their PDA. The game prompted the participants to predict their upcoming BG values, insulin doses, and carbohydrate intake.	• Hyperglycemia.<br>• HbA1c.	Significant difference.	• Game group had significantly less hyperglycemia (glucose ≥13.9 mmol/L or ≥250 mg/dL) than the control group (P < 0.001).<br>• Game group maintained HbA1c values at <8% (P = 0.06).
Leu et al. 2005 [76]	Wireless 2-way pager-based automated messaging system.	• Experimental group (n = 25).<br>• Control group (n = 25).<br>• Study period: 6 months.<br>• Patients with diabetes diagnosis.	Patients did not receive support from the health care team via the pager, but could page the investigator if they had any questions.	Patients in the pager group received instructions on pager use. Reminders, including custom reminders for time of day and reinforcement for other health-related tasks, were sent.	• HbA1c.<br>• BP.	Mixed results.	• Average HbA1c levels decreased in both groups, with average decrease of 0.1–0.3%.<br>• Half as many patients in pager group compared to control group were hypertensive at the end of the study (P = 0.013).

Reference	mHealth tool used	Study design	Control group	Intervention group	Outcome measures	Significance	Results
Lim et al. 2011 [53]	Individualized interactive u-healthcare service based on automated CDSS using SMS on mobile phones.	• Routi ne care (n = 48).<br>• SMB G group (n = 47).<br>• u-healthcare group with wired telephone-connected glucometer plus mobile phone (n = 49).<br>• Study period: 3 months.<br>• Diabet es patients, average age 67-68 years.	• Control group received no intervention; followed regular medical care.<br>• SMBG group: measured their BG level at least 8 times a week.	• u-healthcare group used public switched telephone network-connected glucometers and measured BG at same frequency as SMBG group.<br>• Rec eived SMS on mobile phones from the CDSS rule engine server based on patient's BG testing.<br>• Eval uation messages on BG levels and frequency of BG testing were also sent at weekly and monthly intervals.	• HbA1c.	Significant difference.	The mean HbA1c level was significantly decreased, from 7.861.3% to 7.461.0% (*P* < 0.001) in the u-healthcare group, and from 7.961.0% to 7.761.0% (*P* = 0.020) in the SMBG group, compared with 7.960.8% to 7.861.0% (*P* = 0.274) in the control group.

Reference	mHealth tool used	Study design	Control group	Intervention group	Outcome measures	Significance	Results
Nagrebetsky et al. 2013 [77]	Stepwise self-titration of oral glucose-lowering medication guided by a mobile platform for improving glycemic control in type 2 diabetes patients.	• Mobile phone group (n = 7).<br>• Usual care group (n = 7).<br>• Study period: 6 months.<br>• Type 2 diabetes patients, average age 58 years, taking glucose-lowering medication.	Usual care, including a supportive lifestyle-focused intervention consisting of monthly telephone calls. Medication titration followed the procedures of routine care of patients with diabetes.	Followed a stepwise treatment plan for titration of oral glucose-lowering medication with self-monitoring of glycemia using real-time graphical feedback on a mobile phone and remote nurse monitoring using a web-based tool.	• HbA1c.<br>• Change in oral glucose-lowering medication dosage.	No significant difference.	• Change in HbA1c from baseline to 6 months was –10 (–21 to 3) mmol/mol (–0.9% [–1.9% to 0%]) in the intervention group and –5 (–13 to 6) mmol/mol (–0.5% [–1.2% to 0.6%]) in the control group.<br>• Six out of 7 intervention group patients and 4 of 7 control group patients changed their oral glucose-lowering medication (P = 0.24).

Reference	mHealth tool used	Study design	Control group	Intervention group	Outcome measures	Significance	Results
Quinn et al. 2008 [78]	Mobile phones with WellDoc™ (WellDoc™ Communications, Inc., Baltimore, USA) diabetes management software.	• Intervention group (n = 13).<br>• Control group (n = 13).<br>• Study period: 3 months.<br>• Adults with type 2 diabetes.	Patients had to fax or call in their BG logbooks every 2 weeks to their HCP until values were stabilized or until testing frequency changed by the provider. Provider followed usual standard of care for diabetes management.	Received a OneTouch® Ultra® (LifeScan, Inc.) BG meter, adequate BG testing strips and lancets, and a mobile phone equipped with WellDoc™'s proprietary software. Patients were instructed that their logbooks would be sent electronically to their HCP every 4 weeks, or sooner if needed.	• HbA1c.<br>• Change in medication dosage.	Significant difference.	• The average decrease in HbA1c for intervention patients was 2.03%, compared to 0.68% (*P* = 0.02, one-tailed) for control patients.<br>• Of the intervention patients, 84% had medications titrated or changed by their HCP, compared to the control group (23%; *P* = 0.002).

Reference	mHealth tool used	Study design	Control group	Intervention group	Outcome measures	Significance	Results
Quinn et al. 2011 [45]	Patient coaching and provider clinical decision support via mobile diabetes management software application and a web portal.	• Group 1, usual care (control) (n = 56).<br>• Group 2, coach-only (n = 23).<br>• Group 3, coach PCP portal (n = 22).<br>• Group 4, coach PCP portal with decision support (n = 62).<br>• Study period: 12 months.	Patients in the usual care group received usual diabetes care. Patients in the coach-only group could share their data with their providers if they chose to do so.	The data-only view (Group 3, CPP) allowed providers to access unanalyzed patient data. Group 4 (CPDS) providers had access to analyzed patient data linked to standards of care and evidence-based guidelines.	• Change in glycated hemoglobin levels.<br>• Patient-reported diabetes symptoms.<br>• BP.<br>• Lipid values.	Mixed results.	• The mean declines in glycated hemoglobin were 1.9% in the maximal treatment group and 0.7% in the usual care group, a difference of 1.2% ($P < 0.001$) over 12 months.<br>• Differences were not observed between groups for patient-reported diabetes distress, depression, diabetes symptoms, or BP and lipid levels (all $P > 0.05$).
Rossi et al. 2010 [79]	DID on mobile phone.	• Control, standard education (n = 63).<br>• Intervention, DID approach (n = 67).<br>• Study period: 6 months.<br>• Diabetes patients, average age 36 years.	Patients received standard education on diabetes management.	Patients received training on use of DID and the software was installed on their phone.	• HbA1c.<br>• Fasting BG levels.<br>• Lipid profile.<br>• BP.<br>• Frequency of hypoglycemia.<br>• Frequency of hospitalization.	No significant difference.	• HbA1c reduction was similar in both groups: intervention group from 8.2 ± 0.8 to 7.8 ± 0.8%, and control group from 8.4 ± 0.7 to 7.9 ± 1.1% ($P = 0.68$).<br>• Non-significant differences in favor of intervention group were documented for fasting BG and body weight. No severe hypoglycemic episodes occurred.

Reference	mHealth tool used	Study design	Control group	Intervention group	Outcome measures	Significance	Results
Shetty et al. 2011 [69]	SMS.	• Control group (n = 66).<br>• SMS group (n = 78).<br>• Study period: 12 months.<br>• Ages: 50 ± 8 years.	Received standard care including prescription medications and advice on diet and lifestyle modifications.	Received a SMS message once every 3 days as a reminder to strictly follow the regimen of dietary modification, physical activity, and medication schedules.	• HbA1c.<br>• Fasting BG level.<br>• Two-hour postprandial BG.<br>• Serum total cholesterol.	Mixed results.	• Mean fasting BG levels (185 + 57 mg/dl to 166 + 54; *P* < 0.002) and 2hPG levels (263 + 84 mg/dl to 220 + 67; *P* < 0.002) decreased significantly in the SMS group.<br>• There was no significant difference in the mean HbA1c values in both groups.<br>• Serum total cholesterol decreased significantly in both groups.

Reference	mHealth tool used	Study design	Control group	Intervention group	Outcome measures	Significance	Results
Yoon and Kim 2008 [59]	Nurse education using SMS service on mobile phone.	• Intervention group (n = 25).<br>• Control group (n = 26).<br>• Study period: 12 months.<br>• Patients with type 2 diabetes, average age 46-47 years.	Participants met with an endocrinologist and received customary outpatient care at the clinic during their visit. Recommendations and educational information on diabetes management were offered during the visit.	Participants sent details of SMBG data to website. Recommendations from the researcher and laboratory data were available on the website for participants. In addition, nurses integrated information based on participants' data and sent recommendations back to each patient by SMS service and Internet weekly.	• HbA1c.<br>• Fasting BG levels.<br>• Two-hour post-meal BG.	Significant difference.	• The change from baseline in HbA1c was –1.32 in the intervention group versus +0.81 in the control group.<br>• Two hours post-meal BG had a significantly greater decline in the intervention group after 12 months when compared with the control group (–100.0 versus +18.1 mg/dl).

Reference	mHealth tool used	Study design	Control group	Intervention group	Outcome measures	Significance	Results
Zolfaghari et al. 2012 [70]	Regular SMS messages related to diet, exercise, diabetes medications, and BG monitoring delivered to patients' mobile phones.	• SMS group (n = 39).<br>• Mobile phone group (n = 41).<br>• Study period: 3 months.<br>• Patients with type 2 diabetes, average age 51-54 years.	Received counseling on nature of disease, risk factors, importance of BG monitoring, and reinforcement of diet, exercise, medications, etc., in a phone call.	Received SMS messages with information on diet, exercise, medication intake, BG monitoring, and stress management over mobile phone, around 6 messages per week.	HbA1c.	No significant difference.	• There was no significant change in HbA1c levels between the two groups ($P = 0.227$).<br>• HbA1c levels dropped significantly within both groups post intervention: SMS mean change –1.01% ($P < 0.001$); control group mean change –0.93% ($P < 0.001$).

Cardiovascular diseases (n = 9)

Reference	mHealth tool used	Study design	Control group	Intervention group	Outcome measures	Significance	Results
Blasco et al. 2012 [56]	Web-based TM system connecting self-measurement devices used by patients and care managers via mobile phone text messaging.	• TM group (n = 102).<br>• Control group (n = 101).<br>• Study period: 12 months.<br>• Survivors of ACS, average age 60 years.	Received lifestyle coaching and usual treatment.	Took self-measurements for BP, heart rate, and weight (weekly) and BG and lipids (monthly) with automatic sphygmomanometer, glucose and lipid meter, and used mobile phone to send data following a structured questionnaire. The data were available for cardiologist to review and send individualized advice via SMS.	• BP.<br>• BMI.<br>• CRF.<br>• LDL cholesterol.<br>• HbA1c.	Significant change.	• Patients in TM group were more likely (RR = 1.4; 95% CI 1.1–1.7; *P* = 0.01) to experience improvement in CRF profile compared to control group.<br>• More patients in TM group achieved goal BP (62.1% versus 42.9%; *P* = 0.012).<br>• BMI was significantly lower in TM group (0.77 kg/m^2 versus 0.29 kg/m^2; *P* = 0.005).

Reference	mHealth tool used	Study design	Control group	Intervention group	Outcome measures	Significance	Results
Carrasco et al. 2008 [71]	WAP sessions and SMS services.	• TM group (n = 142).<br>• Control group (n = 143).<br>• Study period: 6 months.<br>• Hypertensive patients, average age 62 years.	Patients followed the same BP monitoring protocol as telemedicine group. Results were recorded on paper and patients interacted with their HCP only during scheduled visits to the office.	Patients sent their self-measured BP, pulse rate, and weight weekly, and responded to questionnaire during each WAP session. Providers accessed the data and could send SMS messages to patients regarding any health-related issue.	BP change.	No significant difference.	Degree of HTN control: TM group 31.7%; control 35.7% (difference: 4.0%, 95% CI –7.0% to 14.9%; $P = 0.47$).
Kiselev et al. 2012 [54]	AACM supported by SMS and mobile phone technology.	• TACM group (n = 102).<br>• AACM (SMS) group (n = 97).<br>• Study period: 12 months.<br>• Hypertensive patients, average age 50 years.	Received consultation with doctor only during clinic visit.	Patient-physician communication carried out by SMS and mobile phone. The system sent regular reminders, was used for collection of data on BP, BMI, and number of cigarettes smoked, and for scheduling patients for office visits.	BP.	Significant difference.	At the end of the year, 77% of patients from the AACM group had achieved goal BP level, more than 5 times higher than the TACM group ($P < 0.001$). The RR of achieving and maintaining goal BP in AACM group was 5.44 (CI 3.2–9.9; $P = 0.005$).

Reference	mHealth tool used	Study design	Control group	Intervention group	Outcome measures	Significance	Results
Koehler et al. 2011 [80]	Mobile phone connected to portable devices for ECG, BP, and weight measurements.	• RTM group (n = 354).<br>• Control group (n = 356).<br>• Study period: 2 years.<br>• Stable chronic heart failure patients, average age 66.9 years.	Received usual care at their physicians without RTM support.	Performed daily self-assessment with ECG, BP device, and weighing scale, and used mobile phone with Bluetooth to transfer data to monitoring center.	• Death from any cause.<br>• Composite of cardiovascular death and hospitalization.	No significant difference.	Compared with usual care, RTM group had no significant effect on all-cause mortality (hazard ratio 0.97; 95% CI 0.67–1.41; *P* = 0.87) or on cardiovascular death or heart failure hospitalization (hazard ratio 0.89; 95% CI 0.67–1.19; *P* = 0.44).
Madsen et al. 2008 [81]	BP readings were registered by PDA and automatically transmitted to server, by which patient-doctor could communicate.	• Intervention group (n = 113).<br>• Control group (n = 123).<br>• Study period: 6 months.<br>• Hypertensive patients, average ages 55-57 years.	Patients were instructed to visit their general practitioner as needed and follow treatment. BP was measured at office.	Patients took their BP measurements, which were then transferred to a central server by a PDA-embedded mobile phone unit. Doctors assessed the measurements on a secure home page. Patients and doctors could communicate by email.	Difference in systolic and diastolic daytime ambulatory BP monitoring change from baseline to follow-up between the intervention and control group.	Mixed results.	• Decrease in systolic daytime ambulatory BP monitoring was –11.9 mmHg in the intervention group and –9.6 mmHg in the control group (mean difference –2.3 [95% CI –6.1 to –1.5]; *P* = 0.225).<br>• After 6 months, more patients in the intervention group reached target BP compared to control (60% versus 38%; *P* < 0.001).

Reference	mHealth tool used	Study design	Control group	Intervention group	Outcome measures	Significance	Results
Morikawa et al. 2011 [55]	Electronic salt sensor and mobile phone used to send emails.	• Intervention group (n = 22).<br>• Control group (n = 19).<br>• Study period: 4 weeks.<br>• Hypertensive patients, average ages 55-57 years.	No intervention other than initial group counseling.	Measured daily salt excretion in overnight urine using the electronic salt sensor. Daily salt excretion was estimated and shown by the electronic salt sensor device. Participants were provided with email through their mobile phones with information about salt content of foods, methods for salt reduction, and a message encouraging a salt-reduced diet.	• BP change.<br>• For the intervention group, changes in daily salt excretion and home BP.	Significant difference.	• SBP decreased by 5.4 mmHg ($P = 0.038$) compared to baseline in the intervention group and by 2.2 mmHg ($P = 0.131$) in the control group.<br>• DBP decreased by 6.2 mmHg ($P < 0.001$) in the intervention group and by 1.6 mmHg ($P = 0.210$) in the control group. The difference in change of DBP was significant ($P = 0.012$).

Reference	mHealth tool used	Study design	Control group	Intervention group	Outcome measures	Significance	Results
Neumann et al. 2011 [19]	Stabil-O-Graph® (I.E.M. GmbH, Stolberg, Germany), a fully automatic, clinically validated BP meter with an upper arm cuff, Bluetooth interface, and Bluetooth-compatible mobile phone.	• Telemetric BP monitoring group (n = 30).<br>• Control group (n = 30).<br>• Study period: 3 months.<br>• Patients with inadequately treated arterial HTN, average ages 55-56 years.	Patients were advised to contact treating physician by phone or visit the office in case of side effects or insufficient BP reduction. Patients received up to 2,300 mg irbesartan in addition to their antihypertensive pre-treatment.	BP data from patients with Stabil-O-Graph® were transmitted from the mobile phone via SMS to the central database server. If alarm criteria were met, an alarm report was generated automatically and sent via email to the physician, who contacted the patient by phone to discuss future treatment strategy. Patients received up to 2,300 mg irbesartan.	BP.	Significant difference.	During treatment, mean SBP showed a more intensive decrease in the telemetric BP monitoring group versus the control group: –17.0 ± 11.1 mmHg versus –9. 8 ± 13.7 mmHg (P = 0.032).

Reference	mHealth tool used	Study design	Control group	Intervention group	Outcome measures	Significance	Results
Quilici et al. 2013 [47]	SMS medication reminder.	• SMS group (n = 250).<br>• Standard care group (n = 249).<br>• Study period: 1 month.<br>• ACS patients, average age 64 years.	After cardiac events, patients were discharged with a prescription of aspirin 75 mg and clopidogrel and were provided with educational sessions highlighting the importance of adherence to recommendations.	After cardiac events, patients received standard care and received a daily personalized SMS.	One-month self-reported aspirin adherence and controlled aspirin adherence using platelet function testing.	Significant difference.	Controlled non-adherent patients assessed by platelet testing accounted for 11.2% of the standard care group versus 5.2% in the SMS intervention group (OR [95% CI]: 0.43 [0.22–0.86]; *P* = 0.01).

Reference	mHealth tool used	Study design	Control group	Intervention group	Outcome measures	Significance	Results
Seto et al. 2012 [20]	Mobile phone-based TM.	• TM group (n = 50).<br>• Standard care group (n = 50).<br>• Study period: 6 months.<br>• Adult patients from a heart function clinic.	The standard care group received standard care, including clinic visits, heart failure education during visits, and the ability to telephone the clinic.	The TM group took daily weight and BP readings and weekly single-lead ECGs, and answered daily symptom questions on a mobile phone over 6 months. Readings were automatically transmitted wirelessly to the mobile phone and then to data servers. Instructions were sent to the patients' mobile phones and alerts to a cardiologist's mobile phone as required.	• Surrogate for heart failure prognosis.<br>• BNP.<br>• LVEF.	Mixed results.	• BNP levels and LVEF improved significantly for both groups from baseline to post study, but did not show a between-group difference.<br>• TM group had significant improvements in BNP from baseline to post study (decreased by 150 pg/mL; $P = 0.02$).<br>• LVEF increased by 7.4% ($P = 0.005$); self-care maintenance increased by 7 points ($P = 0.05$); and management increased by 14 points ($P = 0.03$) in the TM group. Control group did not change.

Chronic lung diseases (n = 6)

Reference	mHealth tool used	Study design	Control group	Intervention group	Outcome measures	Significance	Results
Liu et al. 2008 [62]	Software installed on mobile phones to support exercise and monitor adherence to the program by sending exercise data to website.	• Mobile phone group (n = 24).<br>• Control group (n = 24).<br>• Study period: 12 months.<br>• Patients with moderate to severe COPD, average ages 71-73 years.	Patients followed the same exercise protocol at home as the intervention group, but without the mobile phone program.	Music software with an individualized tempo was installed on the patients' mobile phones. Patients performed endurance exercise training by walking at a speed following the music. The mobile phone recorded the duration of music played, equal to the duration of walking.	• Exercise tolerance.<br>• FEV_1.<br>• Inspiratory capacity.	Mixed results.	• Mobile phone group showed significant improvement in breathlessness as measured by Borg scale, compared to baseline measurement. These changes were not observed in control group.<br>• No significant change in FEV_1 was observed in either group, but inspiratory capacity significantly increased in mobile phone group after 12 weeks and continued to improve until end of study period of 12 months.

Reference	mHealth tool used	Study design	Control group	Intervention group	Outcome measures	Significance	Results
Lv et al. 2012 [40]	SMS reminders and information on asthma management.	• SMS group (n = 30).<br>• Traditional group (n = 27).<br>• Control group (n = 14).<br>• Study period: 12 weeks.<br>• Asthma outpatients, average ages 36-41 years.	• Control group: Participants received verbal asthma education from physicians during clinic visit.<br>• Traditional group: In addition to above asthma education, patients used a free PEF meter.	SMS group: In addition to receiving asthma education, patients received SMS reminders twice daily about how to manage asthma. Patients could also send SMS messages to clinic investigators if they had any questions and needed further guidance.	• FEV_1.<br>• PCA measure using PCAQ-6.<br>• AQLQ.<br>• Pulmonary function.	Mixed results.	• PCAQ-6 scores improved significantly in traditional and SMS groups ($P < 0.001$), whereas control group did not show improvement.<br>• Change in AQLQ scores differed significantly in the three groups ($P = 0.008$), with scores highest in the SMS group.<br>• FEV_1 improved in all groups, and difference was not significant.

Reference	mHealth tool used	Study design	Control group	Intervention group	Outcome measures	Significance	Results
Ostojic et al. 2005 [61]	SMS.	• Study group (n = 8).<br>• Control group (n = 8).<br>• Study period: 16 weeks.<br>• Asthma patients, average age 24.6 years.	One-hour asthma education session with specialist and treatment. Control group noted PEF measurements, medication use, and symptoms in paper diary.	In addition to education, patients in the SMS group were instructed to send their PEF results daily via SMS and received weekly instructions from asthma specialist on adjustments of therapy, and follow-up based on PEF measurements sent.	• Pulmonary function test.<br>• Clinical records.<br>• Patients' daily records of PEF and symptoms.<br>• PEF variability.	Mixed results.	• There was no significant difference between the groups in absolute PEF, but PEF variability was significantly smaller in the study group ($P = 0.049$).<br>• FEV_1 in the study group was slightly but significantly increased (81.25 versus 77.63; $P = 0.014$), and unchanged in the control group (78.25 versus 78.88; $P = 0.497$).<br>• Mean FEV_1 was similar in the two groups both before and after the study.<br>• Control group had significantly higher scores for cough (1.85 versus 1.42; $P < 0.05$) and night symptoms (1.22 versus 0.85; $P < 0.05$).

Reference	mHealth tool used	Study design	Control group	Intervention group	Outcome measures	Significance	Results
Prabhakaran et al. 2010 [82]	SMS.	• SMS group (n = 60).<br>• Control group (n = 60).<br>• Study period: 12 weeks.<br>• Asthma patients >21 years.	Asthma education tailored to individual needs followed by conventional inpatient asthma management.	Asthma education tailored to individual needs followed by asthma management supported by SMS monitoring for 12 weeks.	• ACT.<br>• Use of nebulization.<br>• Emergency department visits.<br>• Hospital admissions for asthma.	No significant difference.	• Improvement in ACT scores in 36 subjects in SMS group compared to 28 in control group, but not statistically significant ($P = 0.113$).<br>• Reduction in number of nebulization and emergency department visits but difference not significant ($P = 0.06$).
Ryan et al. 2012 [27]	The t+ Asthma application for recording and transmission of symptoms, medication use, and peak flow.	• Mobile phone group (n = 145).<br>• Control group (n = 143).<br>• Study period: 6 months.<br>• Adolescents and adults with poorly controlled asthma.	Patients in the control (paper) group were asked to keep a paper diary, recording the same data as the intervention group (symptoms, medication use, and peak flow readings) twice daily.	Patients in the mobile phone group were provided with the t+ Asthma application, which enabled twice-daily recording and transmission of symptoms, medication use, and peak flow. Incursion into the red or amber zones triggered contact by an asthma nurse. Both the patient and their clinician were able to access the patient data.	• AQC.<br>• Adverse occurrences (admissions for exacerbations of asthma, prescriptions for oral steroids, unscheduled consultations).<br>• Prescription of asthma medications.	No significant difference.	• More people in the mobile phone group showed improvement in ACQ, although the difference was not significant.<br>• No significant difference in number of acute attacks, steroid courses, or unscheduled consultation visits was observed between the two groups.

Reference	mHealth tool used	Study design	Control group	Intervention group	Outcome measures	Significance	Results
Strandbygaard et al. 2010 [39]	SMS.	• SMS group (n = 12).<br>• Control group (n = 14).<br>• Study period: 12 weeks.<br>• Patients with clinical history of asthma, ages 18-45 years.	Patients received prescription medications for last 4 weeks of study period, but did not receive any SMS reminders about medication intake.	Patients received the prescribed medications as well as SMS messages daily reminding them to take their asthma medication.	• Change in eNO levels.<br>• FEV_1.<br>• Airway responsiveness.	No significant difference.	Overall improvement in eNO, airway responsiveness, and FEV_1, but not different between groups.
**Diabetes mellitus + cardiovascular disease (n = 4)**							
Earle et al. 2010 [58]	Bluetooth wireless technology and 3G mobile terminals to record and transmit readings.	• TM group (n = 72).<br>• Control group (n = 65).<br>• Study period: 12 months.<br>• Patients with asthma diagnosis, average ages 37-40 years.	Control patients did not receive any mHealth equipment. They were not required to report their BP and did not receive any support from the research nurses.	Patients in the intervention arm were trained to measure their own BP and transmit the recordings via Bluetooth wireless technology to a mobile phone once a week.	• Diabetic retinopathy.<br>• HbA1c.<br>• BP.<br>• Total and HDL cholesterol.<br>• Total triglycerides.	Mixed results.	• SBP decreased significantly in the intervention group (–6.5 mmHg; $P = 0.027$), but remained unchanged in the control group (2.1 mmHg; $P = 0.57$).<br>• No significant changes in DBP, HbA1c, or cholesterol profile in either group.

Reference	mHealth tool used	Study design	Control group	Intervention group	Outcome measures	Significance	Results
Istepenian et al. 2009 [83]	Bluetooth wireless technology and 3G mobile terminals to record and transmit readings.	• TM group (n = 72).<br>• Control group (n = 65).<br>• Study period: 12 months.<br>• Patients with asthma diagnosis, average ages 37-40 years.	Patients in the control group received usual care from their practitioners according to normal standards.	Patients in the intervention arm were trained to measure their own BP and transmit the recordings via Bluetooth wireless technology to a mobile phone once a week.	• SBP.<br>• DBP.<br>• Total and HDL cholesterol.<br>• Total triglycerides.	Mixed results.	• Mean [95% CI] significant fall in SBP in patients in the intervention group (–6.5 mmHg [–0.8 to –12.2]; *P* = 0.027), but not in the control group (2.1 mmHg [9.3 to –5.0]; *P* = 0.57).<br>• There were no significant changes in DBP, HbA1c, or cholesterol profile in either group.
Logan et al. 2012 [60]	Home BP monitoring system using custom software application in a BlackBerry smartphone.	• Self-care group (n = 55).<br>• Control group (n = 55).<br>• Study period: 12 months.<br>• Diabetes patients with uncontrolled systolic HTN.	Participants were taught how to measure BP and asked to measure twice for 2 days per week. They were also provided with information on self-measurement of BP and treatments for HTN. They received usual care from primary care physician.	Patients were taught to use the TM system. Patients were asked to take their smartphones to their physician, show them the summary report, and identify threshold BP values for critical alerts messages.	• Change in daytime ambulatory SBP.<br>• Change in home BP readings.<br>• Psychological questionnaire responses.<br>• HTN medications.	Significant difference.	• Mean daytime ambulatory SBP decreased significantly in the intervention group (by 9.1 ± 15.6 mmHg; *P* < 0.0001), but not in the control group.<br>• The mean between-group difference was 7.1 ± 2.3 mmHg (*P* < 0.005).<br>• More patients in the self-care group achieved the target BP of <130/80 mmHg (51% versus 31% in control group; *P* < 0.05).

Reference	mHealth tool used	Study design	Control group	Intervention group	Outcome measures	Significance	Results
Yoo et al. 2009 [85]	UCDC with BG-measuring device, strips, and lancets. An automatic BP monitoring device and body weight scales.	• Intervention group (n = 57).<br>• Control group (n = 54).<br>• Study period: 3 months.<br>• Overweight patients with type 2 diabetes and HTN.	Patients in the control group visited their clinic according to their routine schedule and received the usual outpatient treatment from their physician during the study period.	UCDC system reminded participants to measure their BG, BP, and weight. The device conducted the BG measurements and sent the results. The UCDC system automatically recorded participants' exercise time. Participants received information via SMS. Physicians could follow trends and send recommendations.	• BMI.<br>• Blood chemistry.<br>• HbA1c.<br>• baPWV.	Significant difference.	• Significant improvements in HbA1c in the intervention group (7.6 to 7.1; $P < 0.001$) compared to the control group (7.4 to 7.6; $P = 0.03$).<br>• Significant reduction in SBP and DBP, as well as improvements in total cholesterol, LDL cholesterol, and triglyceride levels in the intervention group.
